# Toward *in vivo*-relevant hERG safety assessment and mitigation strategies based on relationships between non-equilibrium blocker binding, three-dimensional channel-blocker interactions, dynamic occupancy, dynamic exposure, and cellular arrhythmia

**DOI:** 10.1371/journal.pone.0234946

**Published:** 2020-11-04

**Authors:** Hongbin Wan, Gianluca Selvaggio, Robert A. Pearlstein

**Affiliations:** Global Discovery Chemistry, Computer-Aided Drug Discovery, Novartis Institutes for BioMedical Research, Cambridge, Massachusetts, United States of America; Weizmann Institute of Science, ISRAEL

## Abstract

The human ether-a-go-go-related voltage-gated cardiac ion channel (commonly known as hERG) conducts the rapid outward repolarizing potassium current in cardiomyocytes (I_Kr_). Inadvertent blockade of this channel by drug-like molecules represents a key challenge in pharmaceutical R&D due to frequent overlap between the structure-activity relationships of hERG and many primary targets. Building on our previous work, together with recent cryo-EM structures of hERG, we set about to better understand the energetic and structural basis of promiscuous blocker-hERG binding in the context of Biodynamics theory. We propose a two-step blocker binding process consisting of:

1. The initial capture step: diffusion of a single fully solvated blocker copy into a large cavity lined by the intra-cellular cyclic nucleotide binding homology domain (CNBHD). Occupation of this cavity is a necessary but insufficient condition for ion current disruption.
2. The I_Kr_ disruption step: translocation of the captured blocker along the channel axis, such that:
  - The head group, consisting of a quasi-rod-shaped moiety, projects into the open pore, accompanied by partial de-solvation of the binding interface.
  - One tail moiety packs along a kink between the S6 helix and proximal C-linker helix adjacent to the intra-cellular entrance of the pore, likewise accompanied by mutual de-solvation of the binding interface (noting that the association barrier is comprised largely of the total head + tail group de-solvation cost).
  - Blockers containing a highly planar moiety that projects into a putative constriction zone within the closed channel become trapped upon closing, as do blockers terminating prior to this region.
  - A single captured blocker copy may conceivably associate and dissociate to/from the pore many times before exiting the CNBHD cavity.

Lastly, we highlight possible flaws in the current hERG safety index (SI), and propose an alternate *in vivo*-relevant strategy factoring in:

1. Benefit/risk.
2. The predicted arrhythmogenic fractional hERG occupancy (based on action potential (AP) simulations of the undiseased human ventricular cardiomyocyte).
3. Alteration of the safety threshold due to underlying disease.
4. Risk of exposure escalation toward the predicted arrhythmic limit due to patient-to-patient pharmacokinetic (PK) variability, drug-drug interactions, overdose, and use for off-label indications in which the hERG safety parameters may differ from their on-label counterparts.

## Introduction

As is widely appreciated throughout the pharmaceutical industry, the risk of acquired torsade de pointes arrhythmia (TdP) is proportional to the fractional decrease in the outward repolarizing current (denoted I_Kr_) of the human ether-a-go-go gene product K^+^ channel (hERG) [1–5] due to occupancy of the ion conduction pathway by hERG-blocking drugs. TdP arises at a threshold level of I_Kr_ reduction, which may auto-extinguish spontaneously, or progress to ventricular fibrillation and death [6–13]. The causal relationship between acquired loss of hERG function and TdP was deduced in the late 1990s, resulting in the withdrawal or black box labeling of several implicated drugs [14], together with the implementation of routine hERG safety assessment and monitoring practices throughout the preclinical and clinical stages of pharmaceutical R&D. A surprisingly high prevalence of hERG activity among drug-like compounds was revealed in the process, the molecular causes of which have been investigated over the years with limited success using a number of experimental and *in silico* approaches. Inadvertent hERG blockade arises frequently among screening hits and early leads, which is often reduced but rarely eliminated via trial-and-error chemical optimization. As a result, hERG activity remains a highly problematic liability during the early, preclinical, and clinical stages of drug R&D. The possibility that hERG blockade is only one part of a multi-dimensional ion channel safety problem has been raised by the Comprehensive *in vitro* Pro-arrhythmia Assay (CIPA) initiative [15]. However, because hERG blockade is far more prevalent than that of other cation channels, multi-channel blockade likely accounts for only a subset of TdP cases (TdP is evoked when the total inward-outward current balance is tipped toward the inward direction beyond a threshold level, irrespective of the cause).

Fractional I_Kr_ reduction manifests as a graded prolongation of the ventricular AP duration (APD), mirrored by the QT interval in the electrocardiogram (ECG). Late-stage preclinical hERG safety assessment consists of rising dose ECG studies in dogs or primates aimed at determining the no observed adverse effect QT prolongation (LQT) exposure level, which is necessarily far above the projected human therapeutic free plasma C_max_ (hereinafter referred to as TFPC_max_).

Preclinical hERG safety assessment and mitigation are chicken-egg problems, in which either the maximum safe therapeutic free plasma C_max_ is limited by the maximum achievable hERG IC_50_, or the minimum safe hERG IC_50_ is limited by the minimum efficacious free plasma C_max_. The objective is to maintain a safe “distance” between hERG occupancy at the therapeutic versus arrhythmic exposure levels, allowing for unintended exposure-driven occupancy escalation in the patient population. However, the *status quo* hERG safety index (SI) [16] is based on an entirely empirical model (expressed as the maximum safe free plasma C_max_ ≤ 1/30 hERG IC_50_) that we show in this work possibly suffers from multiple flawed assumptions.

*In vitro* hERG potency is necessarily mitigated to the lowest possible degree (ideally, the limit of detection), constrained by efficacious primary target potency, safe off-target potency, solubility, permeability, and other requisite drug-like properties. Mitigation is typically approached via trial-and-error chemical analoging, guided by *in vitro* testing and *in silico* prediction aimed at achieving a therapeutic index (TI) *in vivo* (i.e. the ratio of the TFPC_max_ to the arrhythmic C_max_ or a designated LQT threshold). However, the hERG TI in humans cannot be predicted reliably in the absence of human pharmacokinetic (PK) data, including the TFPC_max_ and potential for exposure escalation due to patient-to-patient PK variability, drug-drug interactions (DDI), and/or overdose. Instead, preclinical safety assessment is performed using safety indices (SI) that are based on general off-target potency-exposure relationships. Redfern et al. developed the following hERG SI based on potency, clinical PK data (including the highest reported C_max_), and reported TdP cases for 100 marketed drugs (including anti-arrhythmic hERG blockers), ranging from no reported cases to TdP-linked withdrawals [16]:

$$\text{Upper}\;\text{safe}\;\text{human}\;\text{TFP}{\text{C}}_{\text{max}}\leq \;\frac{1}{30}in\;vitro\;\text{hERG}\;\text{I}{\text{C}}_{50}$$ (1)

The Redfern SI implicitly accounts for exposure escalation via a wide 30-fold margin between the *in vitro* hERG IC_50_ and TFPC_max_, which as we demonstrate below, translates to nearly zero tolerated hERG occupancy at the maximum anticipated therapeutic exposure in humans. However, the safety margin for compounds exhibiting residual hERG activity at the projected TFPC_max_ cannot be assessed systematically or tailored to benefit/risk via the all-or-none Redfern criterion. Considerable time and effort may be invested in hERG mitigation to the limit of detection, which is subject to the following caveats:

1. Constraints on chemical mitigation imposed by the typically high overlap among the structural and physico-chemical properties promoting hERG and primary target potency, solubility, permeability, and PK behaviors.
2. Insufficient accuracy of *in vitro* assays (typically, radio-ligand displacement and automated or manual patch clamp) needed to resolve true hERG structure-activity relationships (SAR). *In vitro* hERG IC_50_ values were shown to vary as a function of cell culture conditions, patch clamp protocol, data fitting approach [17–19], and temperature [18]. IC_50_ variation of 16- and 23-fold has been reported for terfenadine and loratadine, respectively [17].
3. The limited relevance of *in vitro* hERG binding/blockade measurements to non-equilibrium conditions *in vivo*.
4. The lack of *in vivo* pro-arrhythmia assessment during the lead optimization stage, which is typically reserved for late-stage clinical candidates (relegating the SI to a prediction of *in vivo* behavior).

In our previous works:

1. We studied dynamic hERG blockade by compounds that are trapped within closed channels (“trappable” blockers) versus those that are expelled during closing (“non-trappable” blockers) using a version of the O’Hara-Rudy model of the undiseased human ventricular cardiomyocyte [20] into which we introduced a Markov hERG blocker binding schema [21]. We showed that blockade by non-trappable blockers builds and decays in tandem with channel opening and closing, respectively, whereas trappable blocker occupancy accumulates with increasing exposure up to the free C_max_.
2. We derived a general analytical treatment of non-equilibrium binding that accounts for binding site buildup and decay cycles driven by translocation, conformational changes, or synthesis and degradation of the binding partners [22]. We showed that binding under dynamic conditions is characterized by time-dependent occupancy (rather than static equilibrium occupancy/potency), which is governed by k_on_ relative to the rate of binding site buildup, concentration/exposure, and k_off_ relative to the rate of binding site decay. Non-trappable hERG blocker binding clearly falls at the extreme end of the non-equilibrium spectrum, given that the open (blocker-accessible) state of hERG normally builds and decays over a 350–400 ms time window [21].
3. We hypothesized that hERG binding is energetically-driven largely by solvation free energy (the putative origin of all non-covalent binding free energy barriers [21,23–27]). Toward that end, we studied the solvation properties of the hERG pore using WaterMap and a homology model of the protein [28] (prior to the publication of the cryo-EM structure of open hERG state). The results of our calculations suggest that the pore lumen is solvated almost exclusively by bulk-like and hydrogen bond (H-bond) depleted water, consistent with low blocker association cost (i.e. no or low de-solvation cost of the pore) and high blocker dissociation cost (i.e. high re-solvation cost of the pore). Association and dissociation costs are therefore plausibly relegated largely to blocker de-solvation and pore re-solvation costs, respectively [21]. Additionally, the association rate is plausibly enhanced by electrostatic interactions between basic blockers and the negative field within the pore.
4. We showed that k_off_ of non-trappable blockers that is slower than the closing rate in dynamic channels is “hijacked” by the closing rate. Since hERG patch clamp assays are typically run at sub-physiological gating frequencies (and gating is neglected altogether in radio-ligand binding assays), this hijacking effect is likely underestimated to varying degrees (i.e. channel closing likely dominates over the re-solvation costs of the dissociated pore and blocker).

Here, we use a theory-guided approach [22] to decipher the structural basis of hERG blockade (including trappability) at the atomistic level, and revisit hERG safety assessment in an *in vivo*-relevant context.

## Materials and methods

We emphasize the knowledge-, rather than computation-driven, underpinnings of this work, which is based on a theory (referred to as Biodynamics) that we described previously [22,27]. Briefly, occupied intra- and inter-molecular states are considered to build and decay in a time-dependent (i.e. non-equilibrium) fashion governed by the rates of binding partner or binding site buildup and decay, together with k_on_ and k_off_ (inter-molecular) or k_in_ and k_out_ (intra-molecular), rather than equilibrium binding metrics (e.g. ΔG, K_d_, K_i_, IC_50_, EC_50_) that pertain primarily to the *in vitro* setting [21,22]. Furthermore, the barriers underlying k_on_, k_off_, k_in_, and k_out_ under aqueous conditions are attributed predominantly to de-solvation and re-solvation costs. In our previous work, we simulated dynamic hERG-blocker occupancy non-atomistically, within the overall context of the cardiac AP, in which the dynamic hERG state transitions vis-à-vis blocker k_on_, k_off_, and concentration were considered explicitly [21]. In this work, we characterize hERG blocker binding at the atomistic level, aiming to qualitatively predict the energetic driving force and canonical binding modes of both trappable and non-trappable compounds.

**Fig 1:**
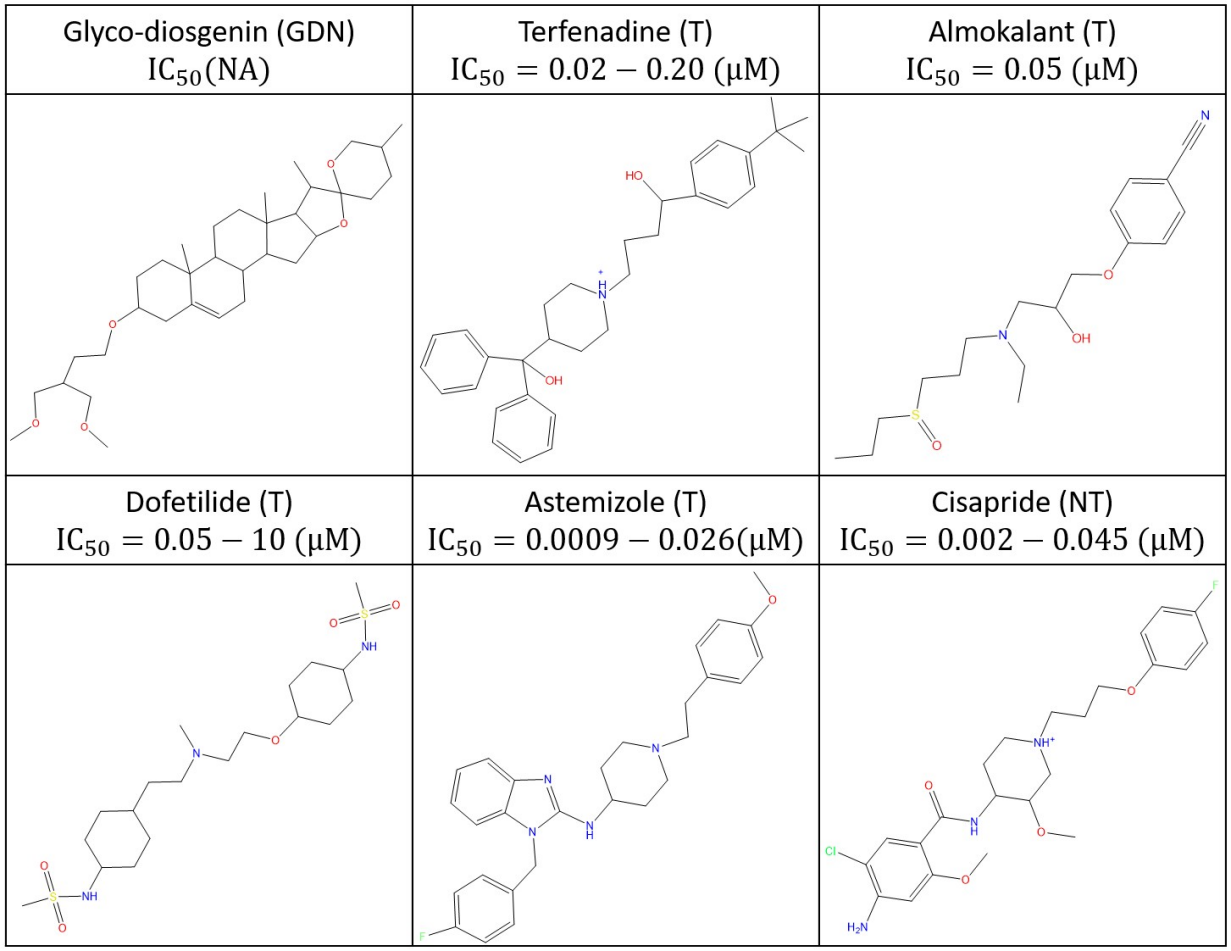
Reference compounds studied in this work, including hERG blockers [16] and GDN [31].

**Fig 2:**
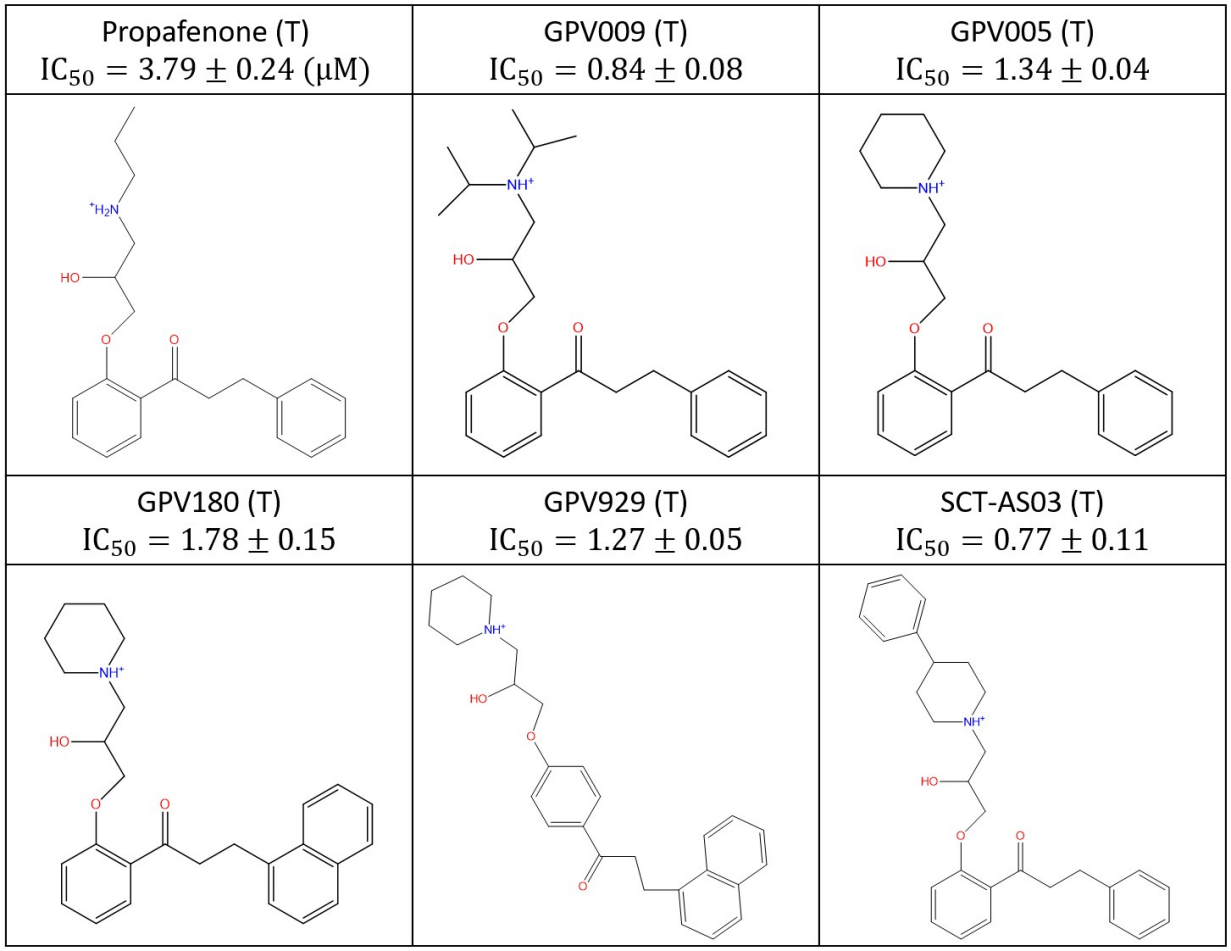
Published trappable propafenone analogs studied in this work [29].

**Fig 3:**
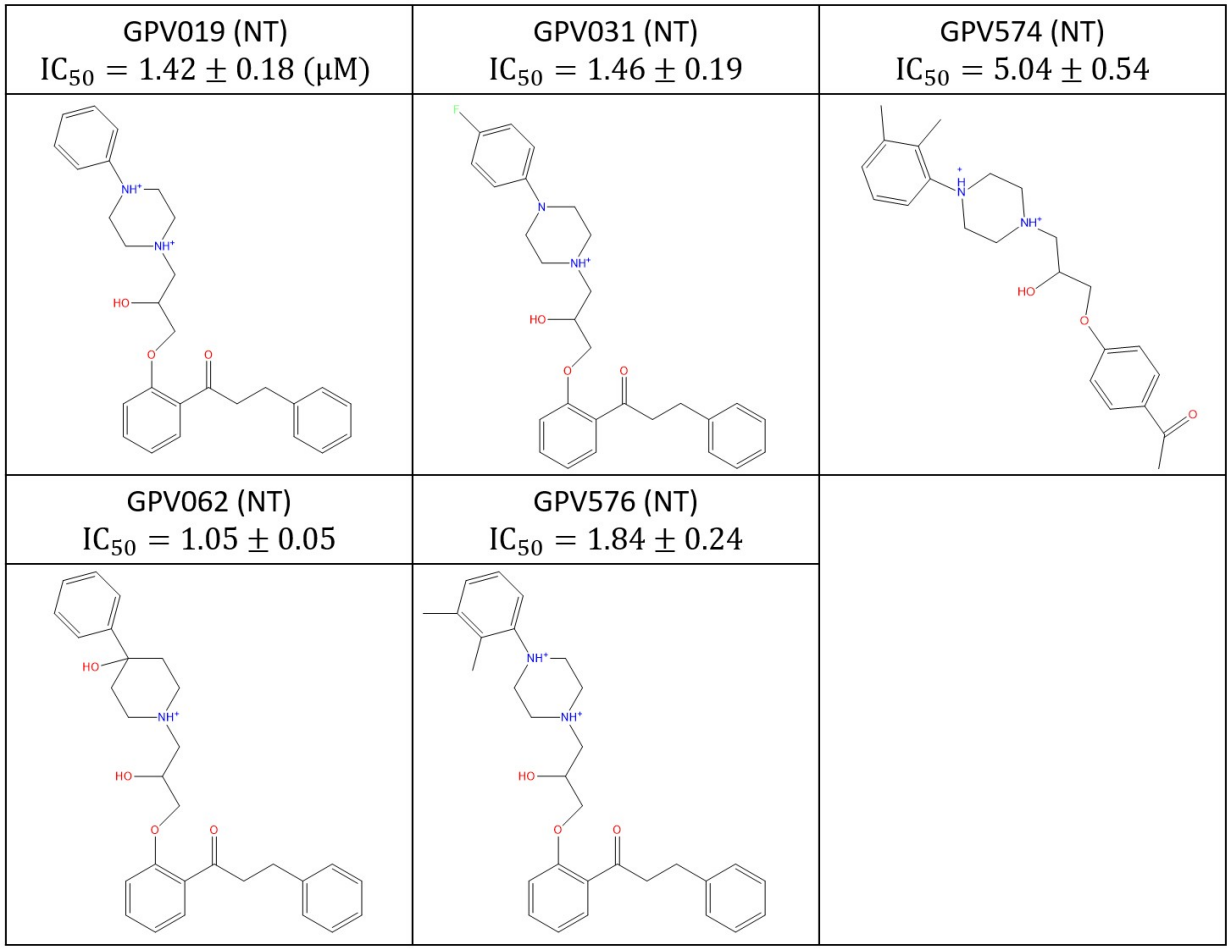
Published non-trappable propafenone analogs studied in this work [29].

All calculations and visualizations were performed using Maestro 2019–1 (Schrodinger, LLC, Portland, OR) on a representative set of canonical hERG blockers taken from reference [16] (Fig 1), as well as trappable and non-trappable propafenone analogs taken from reference [29] (Figs 2 and 3, respectively). The structures were built using LigPrep and energy minimized using MacroModel (MMFF force-field and default parameters). The hERG structure (PDB codes = 5VA1, 5VA2 [30]) was prepared (hydrogen addition, Asn, Gln, His orientations) using PPrep. Blocker docking sites and the solvation properties thereof were characterized using SiteMap. The cryo-EM structure of Na_v_1.4 (PDB code = 6AGF [31]) in complex with a glyco-diosgenin (GDN) detergent molecule, the rigid rod-like and bulky flexible disaccharide moieties of which straddle between the pore and cytoplasm, respectively. This finding suggests a possible canonical binding motif for many Y and L-shaped blockers containing similar rod-like and bulky moieties that bind within the intra-cellular pore segment of many voltage-gated ion channels. To test this hypothesis, we used the pore-bound region of GDN as a template on which we superimposed the hERG blockers in our study heuristically (rather than computationally) in quasi-extended conformations in order to generate a common superposition reference geometry, rather than to predict the specific bound conformations of the blockers (noting that a common superposition could not be achieved in the putative outer vestibule-bound region due to the greater chemical and structural diversity in this region).

## Results

We characterized the energetic, structural, and chemical drivers of hERG blocker binding and trappability using modeled three-dimensional structures of known trappable and non-trappable compounds, together with a set of recently published cryo-EM structures of the full length open hERG channel (PDB codes = 5VA1, 5VA2 [30]), and the closed EAG1 channel (PDB code = 5K7L [32]). Our overall findings suggest that blocker binding is governed by the following contributions:

1. Steric shape and size complementarity between blockers and the pore-bound region.
2. Blocker k_on_ (proportional largely to the blocker de-solvation free energy cost) vis-à-vis the channel opening rate, together with blocker k_off_ (proportional to the protein and blocker re-solvation free energy costs) vis-à-vis the channel closing rate.
3. Blocker basicity/pKa vis-à-vis the negative field within the pore, which speeds the association rate.

Next, we outline an *in vivo*-relevant hERG mitigation strategy based on these findings. Lastly, we revisit the *status quo* hERG safety assessment protocol, and propose an *in vivo*-relevant strategy centered on the putative relationship between dynamic hERG occupancy, PK, and cellular arrhythmogenesis.

### Blockade-relevant aspects of hERG structure and function

**Fig 4:**
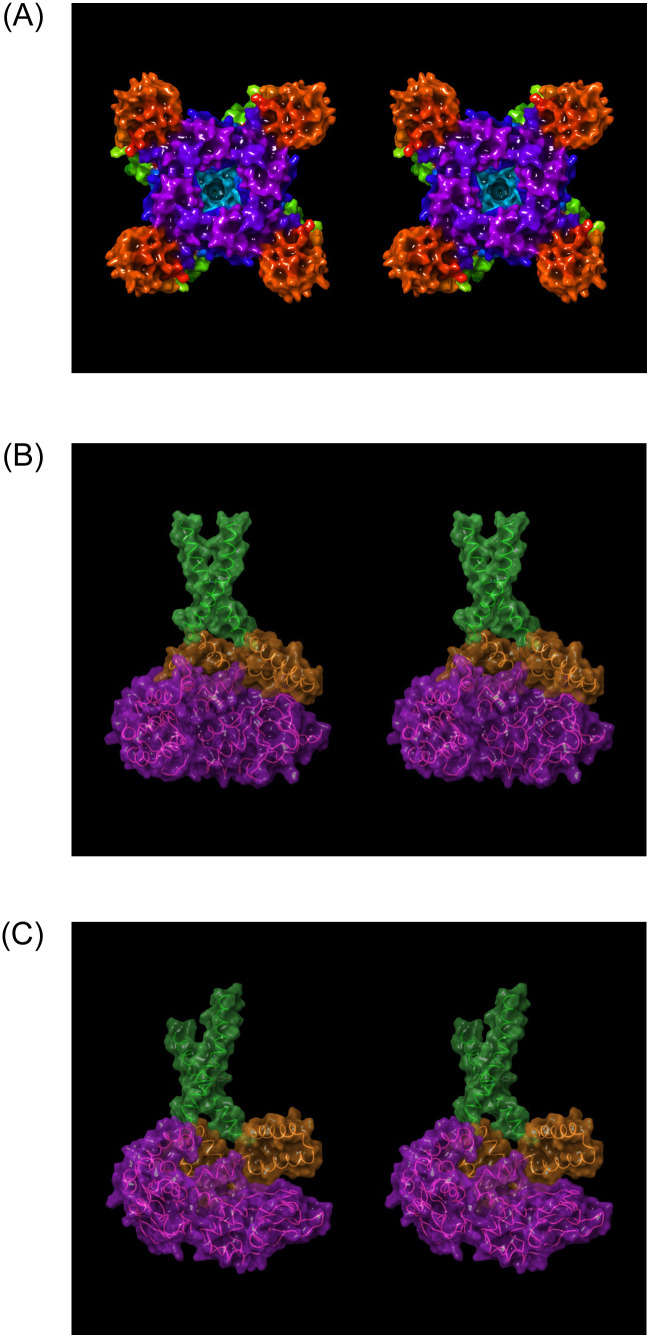
(A) Stereo image of the open state hERG cryo-EM structure (PDB code = 5VA1 [30]) viewed along the pore axis from the intra- to extra-cellular direction. The pore (highlighted in cyan) and CNBH domain (highlighted in purple) cavities reside at the distal and proximal ends of the structure, respectively. The helical C-linker domains (highlighted in blue), with the voltage-sensing domains (highlighted in light green) are partitioned above in the membrane. The C-linker domains reside between the pore and CNBH domains, forming a continuous intra-cellular cavity with the latter (referred to hereinafter as the “outer vestibule”). The PAS domain (reddish-orange) resides at the corners of the protein. (B-C) Longitudinal cutaway views of the intra-cellular region of the ion conduction pathway, consisting of the C-linker lined cavity (highlighted in orange), which is sandwiched between the intra-cellular pore entrance (highlighted in green) and CNBH domain cavity (highlighted in magenta).

The hERG channel is a tetrameric protein comprised principally of Per-Arnt-Sim (PAS), transmembrane voltage sensing, transmembrane pore, C-linker, and intra-cellular cyclic nucleotide binding homology (CNBH) domains (one per monomer) [33] (Fig 4). We hypothesize that the CNBHD serves as a tetramerization domain, the dissociation of which is slowed by unfavorable re-solvation at H-bond depleted positions within the inter-subunit interface [27] (noting that the absence of this domain in Na_v_1.5 and Ca_v_1.2 is consistent with the single chain composition of these channels). Deletion of the CNBH domain was, in fact, shown to result in the loss of functional tetramers [34]. A large intra-cellular tunnel-like cavity within the combined CNBH/C-linker domains (referred to hereinafter as the “outer vestibule”) is observed in the cryo-EM structure, through which blockers must necessarily transit in accessing the pore.

### The predicted canonical binding mode of voltage-gated ion channel blockers

It is widely assumed that bound hERG blockers are fully buried within the pore domain based on mutagenesis data and *in silico* docking [35,36], which we had likewise assumed in our previous work [28]. However, this assumption leads to the following potential caveats:

1. Excessive induced fit levels and unfavorable folded conformations needed to fully accommodate blockers whose volume exceeds that of the relaxed pore (noting that the quasi-extended conformations of most blockers are too large to fit longitudinally within the pore). Furthermore, translocation from the outer vestibule into the pore depends in many cases on the passage of bulky blocker groups through the intra-cellular pore entrance (the width of which is governed by Gln664).
2. The lack of a straightforward explanation of trappability, which as we postulate below, is governed by the specific nature of blocker groups projecting through a constriction zone that exists in the closed state of the pore (or avoidance of this zone completely).
3. A complex binding mechanism follows from this scenario, in which:
  - One blocker copy occupies the pore, and the other occupies the outer vestibule.
  - The pore-bound copy is trapped transiently by the outer vestibule-bound copy, such that channel closing is either hampered, or both copies are expelled simultaneously during channel closing.

**Fig 5:**
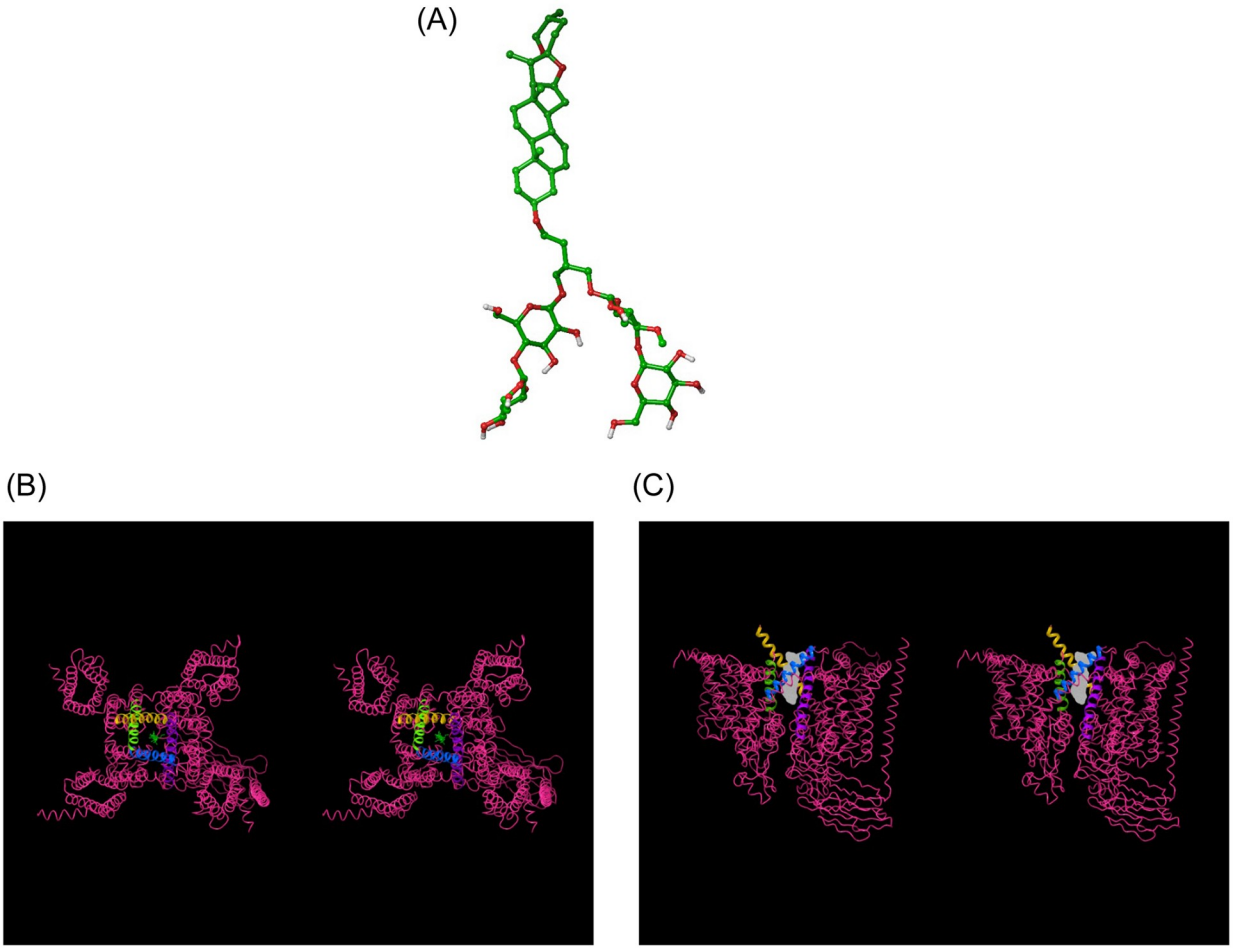
(A) The structure of GDN (PDB code = 6AGF [31]), in which the unresolved disaccharide moieties residing at the bottom of the structure were qualitatively modeled in. (B) The Na_v_1.4 channel viewed parallel to the pore axis from the intra- to extra-cellular direction, showing the GDN molecule (green) bridging between the pore (which is partially closed) and cytoplasm (corresponding to the C-linker-enclosed cavity in hERG). (C) Same as B, except viewed perpendicular to the pore axis, with the cytoplasmic end of the channel at the top of the figure. The molecular surface of GDN is shown in gray.

A glyco-diosgenin (GDN) detergent molecule (Fig 5A) bound to the closed state of the human voltage-gated Na_v_1.4 channel, observed in a recent cryo-EM structure (PDB code = 6AGF) [31], offers a possible clue as to the general binding mode of many cation channel blockers (noting that significant disruption of the S6 helix is observed in the presence of this molecule). GDN straddles the pore and pore entrance with its rod-like polycyclic moiety buried within, and its two unresolved disaccharide moieties projecting out to the cytoplasm (reminiscent of a “drain-plug”) (Fig 5B and 5C). We proceeded to test whether hERG blockers could potentially bind according to a similar drain-plug paradigm using a ligand-based overlay model that we manually docked in the hERG cryo-EM structure (noting that the intra-cellular hERG blocker moiety would necessarily reside within the outer vestibule, rather than the cytoplasm).

**Fig 6:**
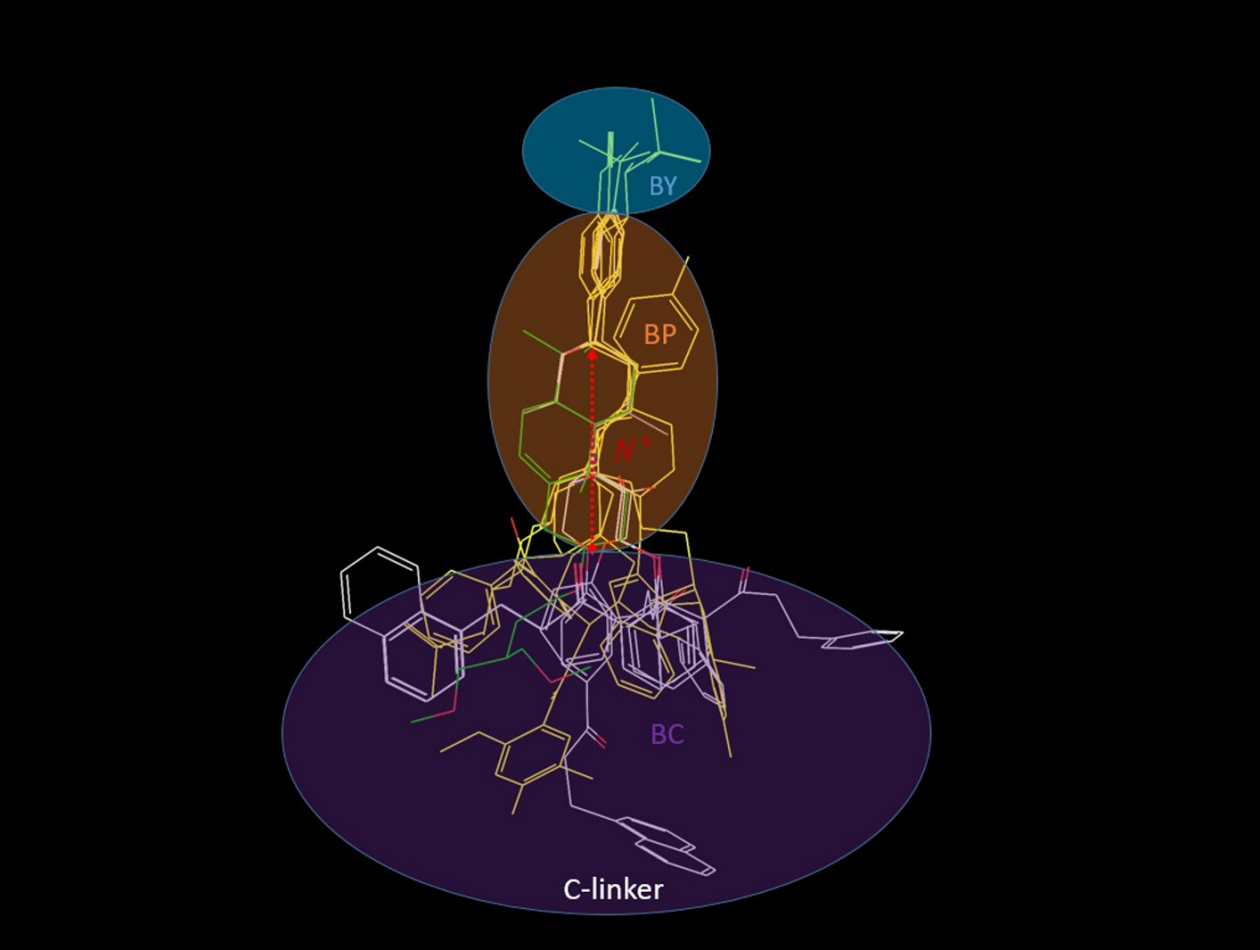
Overlay of the reference set of blockers (see Materials and methods) in the proposed canonical binding mode, which is similar to the CoMFA model reported by Cavalli et al. [37]. BP consists of diverse quasi-rod-shaped or mildly kinked blocker moieties, typically consisting of one or more hetero-atom containing planar/aromatic or saturated rings, which may be fused (e.g. spiro) or connected by linkers. BC likewise consists of diverse substructures (which do not converge to a common conformation), including butterfly-shaped bisaryl groups.

**Fig 7:**
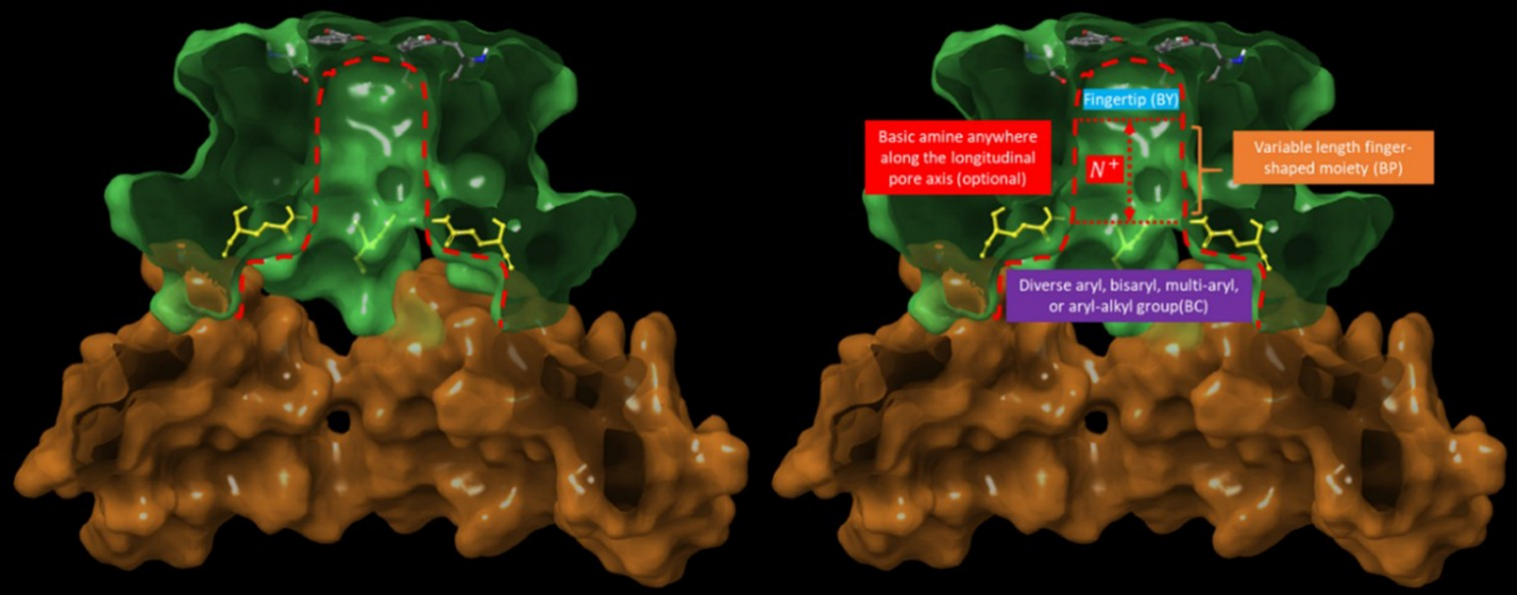
Longitudinal cutaway of the pore and funnel-shaped C-linker cavities (highlighted in green and brown, respectively), showing Tyr652 (comprising the proposed Y docking site) residing at the top of the pore, proximal to the intra-cellular face of the selectivity filter, and Gln664 (highlighted in yellow). Gln664 lines the entrance of the open pore, restricting the translocation of BC into the pore. The pore (enclosed within the red dotted outline) is shown without and with annotations for clarity (left and right panels, respectively).

We overlaid the hERG and Na_v_1.4 cryo-EM structures, and manually fit our reference set of hERG blockers (see Materials and methods) to the common pore-bound moiety of GDN (Fig 6). According to our model, the typically Y- or L-shaped hERG blockers project a single quasi-rod-shaped moiety into the pore. We assume that basic nitrogen-containing moieties, when present, reside within this region. Our analysis suggests the existence of three primary blocker-hERG docking interfaces within the ion conduction pathway (Fig 7), as follows:

1. The region of the lumen enclosed by the four two-helix bundles of the C-linker residing adjacent to the intra-cellular pore entrance (denoted as “C”). L- and Y-shaped blockers likely project moieties (denoted as “BC”) into this region, whereas linear blockers may not.
2. The pore lumen spanning between the intra-cellular entrance and intra-cellular face of Tyr652 (denoted as “P”). Blockers project a single quasi-rod-shaped moiety (denoted as “BP”) into this region. The occupied distance along the pore axis and angle of the rod-shaped blocker moiety relative to the pore axis vary among the blockers in our study (Fig 3) (noting that steric compatibility between the blocker and pore likely depends on limited angular deviation of the long axis of BP from that of the pore).
3. The upper region of the pore adjacent to the side chains of Tyr652 (denoted as “Y”). Blockers project the terminal BP group into this region (denoted as “BY”), noting that only a subset of blockers terminate in this region (i.e. those that maximally occupy P).

**Fig 8:**
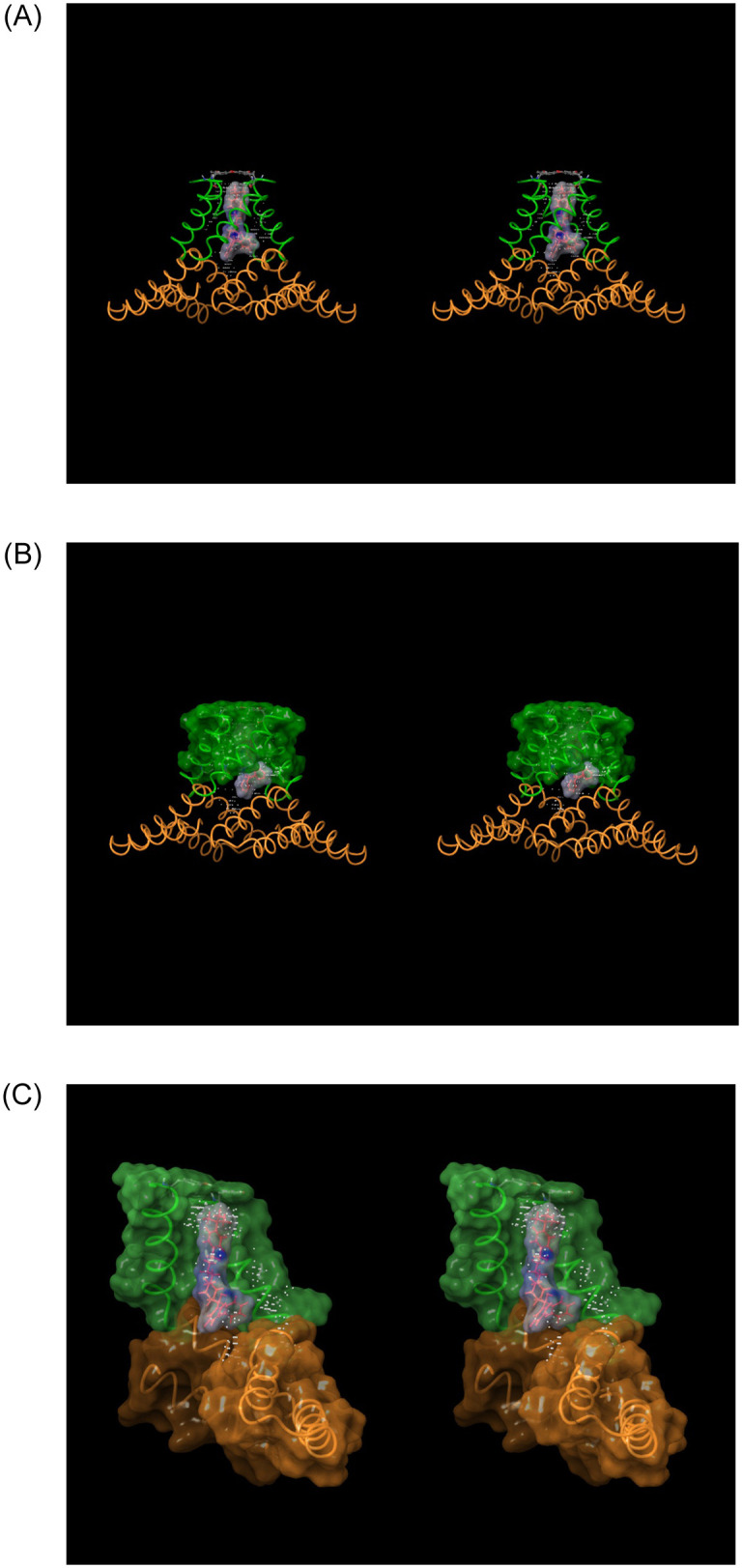
(A) Stereo image of the proposed canonical hERG binding mode (exemplified by terfenadine, shown as a molecular surface), in which blockers straddle between the Y and C regions of the pore (where the C region is comprised of the kink between the S6 and proximal C-linker helices). (B) Same as A, but showing the putative protrusion of the tail region of terfenadine from the pore domain (green surface) into the C-linker-enclosed portion of the outer vestibule. (C) Terfenadine was docked manually into clusters of SiteMap site points (white spheres) described in Materials and methods. The butterfly-shaped diphenylmethane tail moiety of terfenadine is complementary in shape to the C-linker helix (noting that butterfly-shaped bisaryl groups are relatively commonplace among hERG blockers [21]).

We manually docked terfenadine in the proposed binding mode (Fig 8A) guided by SiteMap site points (the white spheres in Fig 8).

**Fig 9:**
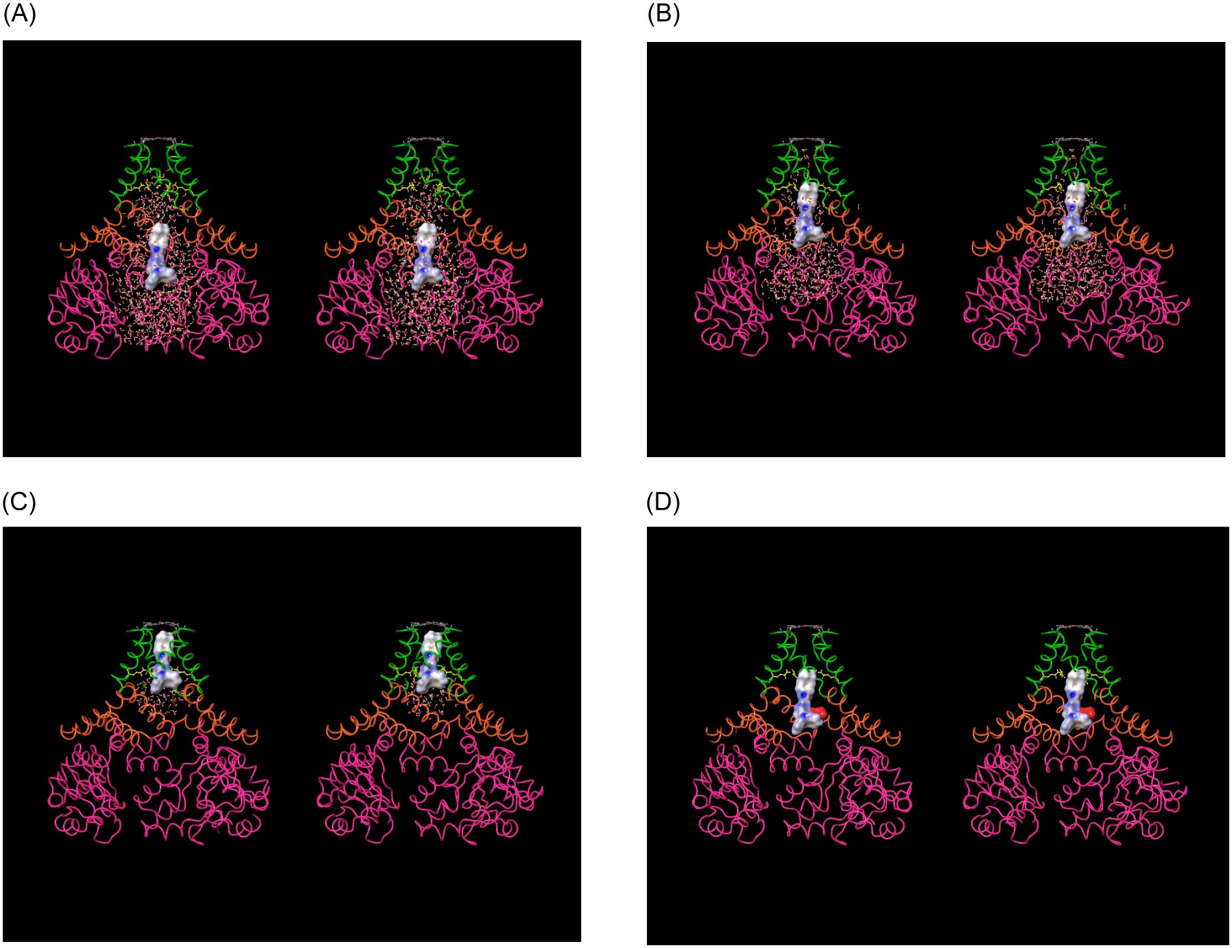
Stereo images of terfenadine manually docked within the cryo-EM hERG structure (PDB code = 5VA1 [30]) at various hypothetical stages of binding (and de-solvation), viewed along the pore axis in the intra- (bottom) to extra-cellular (top) direction. (A) Fully solvated terfenadine, manually docked within the CNBH domain region of the outer vestibule prior to putative repositioning of BC, BP, and BY into their corresponding C, P, and Y docking sites. Blockade of I_Kr_ due to occupancy of the outer vestibule is unlikely. (B) Terfenadine manually docked with its partially de-solvated BP region penetrating above the pore entrance. (C) Terfenadine manually docked in its hypothetical final binding mode, with the fully de-solvated BC, BP, and BY regions in contact with the C, P, and Y docking sites, respectively. Four such configurations are conceivable due to the 4-fold symmetry of the channel. (D) Cartoon depicting the putative H-bond enriched solvation of BC (red surface). The degree of H-bond enrichment determines the maximum de-solvation free energy cost (the barrier to translocation of BC into C, which behaves like a “bumper” between the two entities in the absence of optimal H-bond replacements).

Blockers necessarily translocate into the pore via the outer vestibule, relegating hERG blockade to a two-step process consisting of:

1. A capture step, in which a single solvated blocker copy diffuses from the cytoplasm into the CNBH domain cavity (which in and of itself is unlikely to block ion conduction into and through the pore).
2. A longitudinal translocation step, in which the captured blocker copy shifts from the CNBHD cavity into the C-linker cavity and pore entrance (Fig 9A and 9B), while simultaneously:
  - Projecting BP into the open state of P, accompanied by full or partial mutual de-solvation of P and BP. Basic groups (when present) reside on BP. BY, the terminal group of BP projects into Y, accompanied by mutual de-solvation of BY and Y. The extent to which BP penetrates into the pore is determined by its length relative to that of P, or the steric size of BY relative to the diameter of the pore entrance.
  - Positioning BC into C, accompanied by full or partial mutual de-solvation of C and BC (noting that BP insertion and BY binding are interdependent processes, given that the two moieties are bonded directly). Putative H-bond enriched solvation of BC is represented as a red “bumper” in Fig 9C. BC is restricted to the C-linker cavity via steric clashing with side chains at the pore entrance (color-coded yellow in Fig 9), and as such, blockers containing BP moieties shorter than the longitudinal pore length necessarily terminate below the Y docking site.

The total mutual de-solvation costs of C-BC, P-BP, and Y-BY putatively serves as the overall blocker association barrier. The results of our previous WaterMap calculations suggest that P is solvated almost entirely by bulk-like and H-bond depleted water, thereby avoiding disruption of the negative field stemming from ordered H-bond enriched water [21]. The outer vestibule was omitted in our WaterMap calculations, which were performed prior to determination of the cryo-EM structure of hERG. H-bond depleted solvation is localized to the non-polar side chains of the pore, and most notably the intra-cellular facing surface of Tyr652 (the Y docking site) located adjacent to the selectivity filter at the distal end of P. We used SiteMap to characterize the solvation within the pore of the cryo-EM structure. As expected, the results are consistent with those of our previous WaterMap calculations [21].

### Proposed blocker structure-kinetics relationships

We proposed previously that non-covalent association and dissociation free energy barriers consist principally of H-bond enriched and depleted solvation free energy (relative to the free energy of bulk solvent), respectively [27]. The rate of blocker association depends on the total solvation free energy of H-bond enriched water expelled from the binding interface (i.e. the mutual blocker-channel de-solvation cost). The rate of blocker dissociation depends on the magnitude of the total free energy cost of re-solvating H-bond depleted positions within the dissociated binding interface (i.e. the total blocker and channel re-solvation cost). H-bond enriched solvation incurs zero re-solvation cost during dissociation, whereas H-bond depleted solvation incurs zero de-solvation cost during association. In our previous work, we demonstrated that the pore in hERG is solvated almost exclusively by H-bond depleted and bulk-like water [21] corresponding to low de-solvation and high re-solvation costs, respectively. The rate of non-trappable blocker binding, therefore, depends largely on the de-solvation cost of the pore-binding blocker moiety, and the dissociation rate is proportional to the channel-closing rate or blocker dissociation rate, whichever is faster (minimally ~2 s^-1^, which we refer to as the “k_off_ floor” [21]). The rate of pore insertion by trappable blockers is likewise proportional to the de-solvation cost of the pore-binding moiety, whereas the k_off_ floor is ~0.7 s^-1^ [21]. It is therefore apparent that, under physiological conditions, dynamic blocker occupancy is influenced heavily by the rates of channel opening and closing, which are typically sub-physiological in patch clamp assays, and always zero in radio-ligand displacement assays (where the channels are static). As such, binding measurements performed under non-physiological conditions do not translate reliably to the *in vivo* setting for non-trappable blockers exhibiting k_off_ < the floor and k_on_ < the rate of channel opening. Percent inhibition in all such cases is increasingly overestimated as k_on_ decreases relative to the channel-opening rate (see [21,22]). Blocker binding kinetics can be qualitatively inferred from conventional structure-activity relationships (neglecting channel-gating dynamics), as follows:

1. Significant decrease in the percent inhibition/occupancy of a given blocker analog resulting from:
  - k_on_ slowing due to increased blocker de-solvation cost incurred during association via the addition of (or increased polarity of) a polar BC, BP, or BY blocker group (especially BY). Examples of reduced hERG activity putatively due to increased polarity are available in [38] and [39].
  - k_off_ speeding due to decreased blocker re-solvation cost during dissociation via the deletion of (or decreased polarity of) a polar BC, BP, or BY blocker group (especially BY).
  - Slowed k_on_ due to reduced pKa of a basic group (or removal thereof) within the BP moiety.
2. Significant increase in the percent inhibition of a given blocker analog, resulting from:
  - k_on_ speeding due to decreased blocker de-solvation cost incurred during association via the deletion of (or decreased polarity of) a polar BC, BP, or BY blocker group (especially BY).
  - k_off_ slowing due to increased blocker and/or hERG re-solvation cost during dissociation via the addition of (or decreased polarity of) a non-polar blocker group (especially in BY).
  - k_on_ speeding due to increased pKa of a basic group (or addition thereof) within the BP moiety.

**Fig 10:**
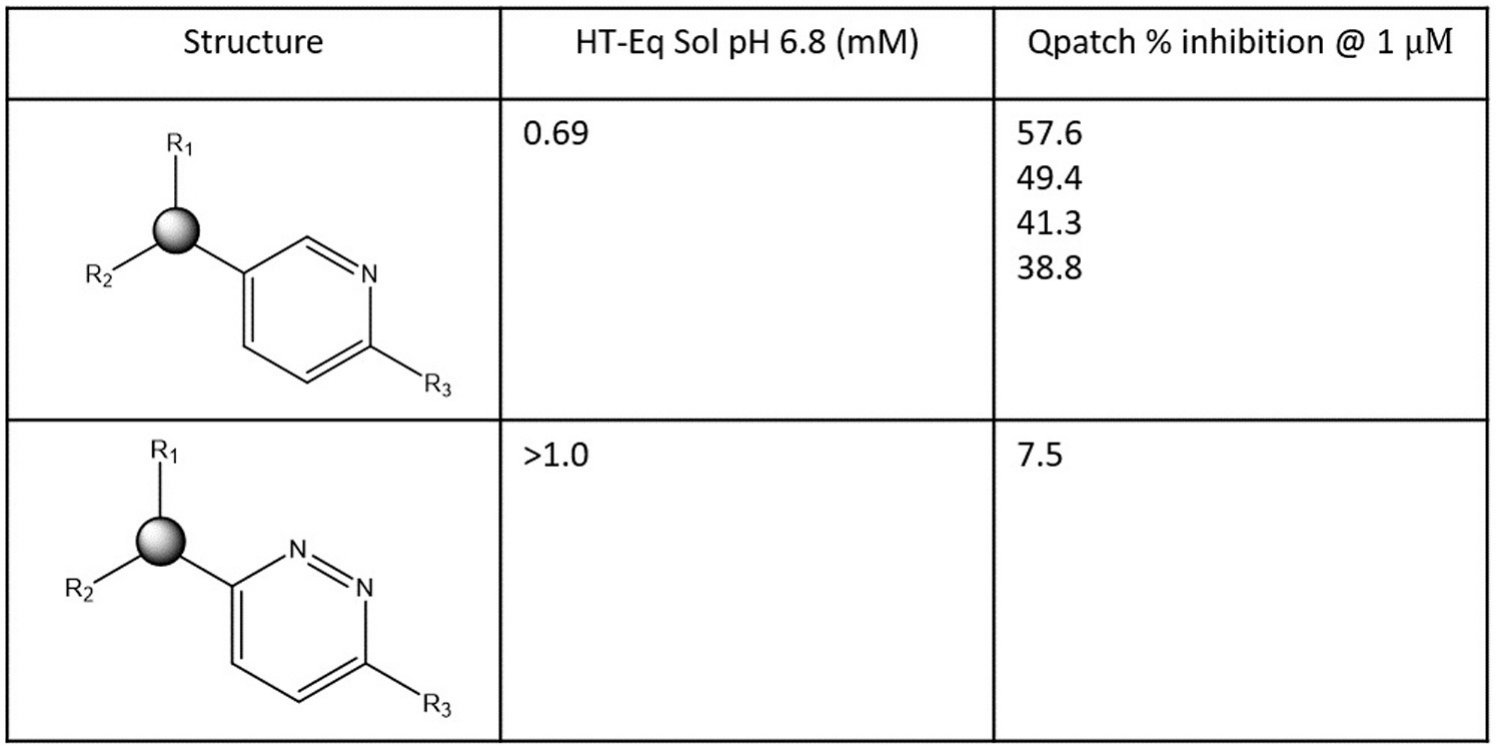
Representative activity cliffs for a proprietary hERG blocker series differing solely in the 6-ring substructures (residing within the putative BC feature set). The large decrease in percent inhibition @ 1 μM for structure 2 compared with structure 1 is attributable to increased polarity and solubility/de-solvation cost of the pyridazyl versus the pyridyl group (consistent with the lack of hERG-contributed H-bond replacements for the water solvating this moiety in the unbound state).

**Fig 11:**
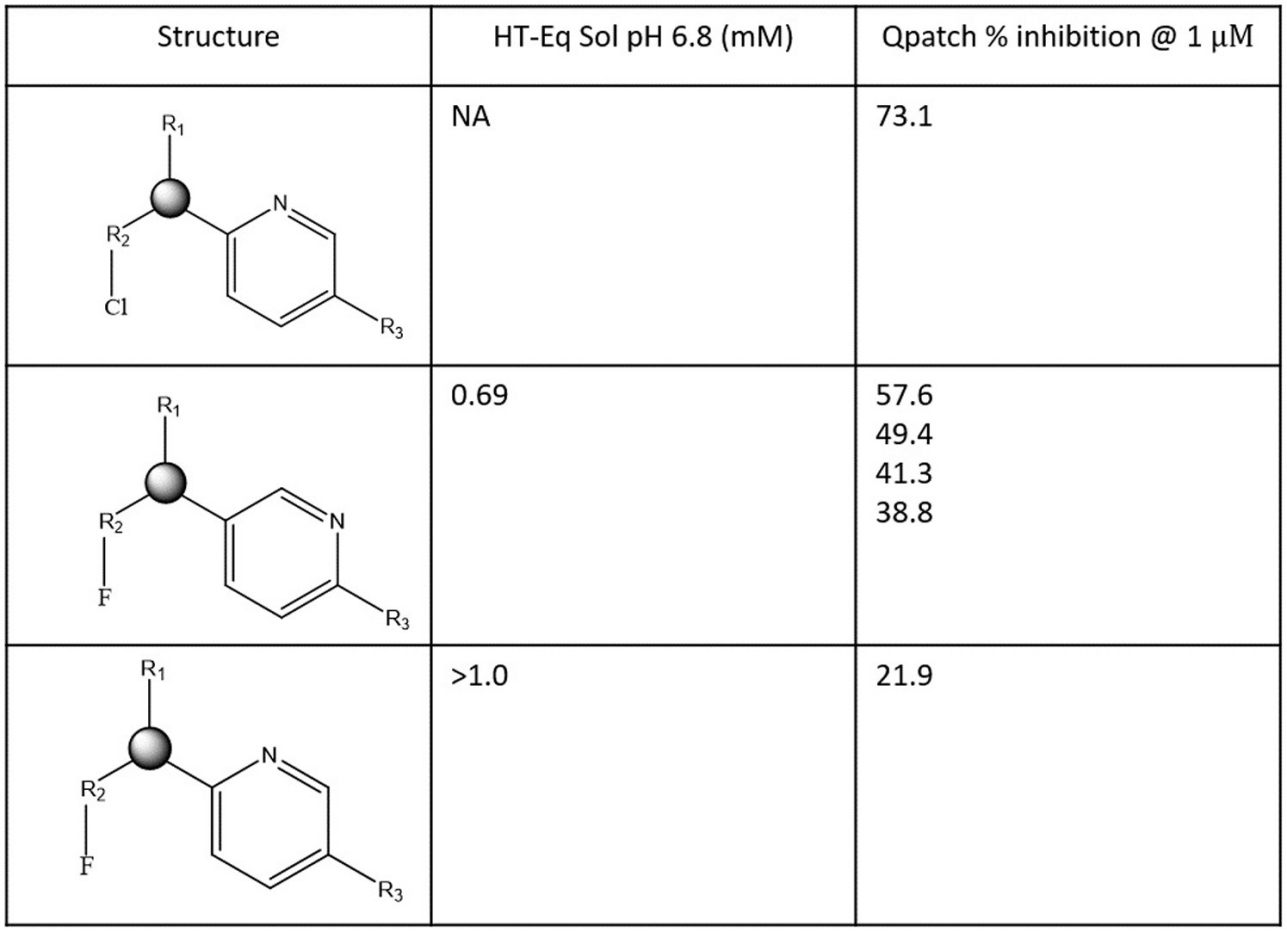
Representative activity cliffs for the proprietary hERG blocker series 1 (see text) comprised of R1 (corresponding to BP) and R2/R3 (corresponding to BC). The large decrease in percent inhibition @ 1 μM for structure 3 compared with structures 1 and 2 is putatively attributable to the greater re-solvation cost of Cl versus F, together with increased polarity and solubility/de-solvation cost of the ortho- versus meta-pyridyl nitrogen, (consistent with the lack of hERG-contributed H-bond replacements for the water solvating this moiety in the unbound state).

**Fig 12:**
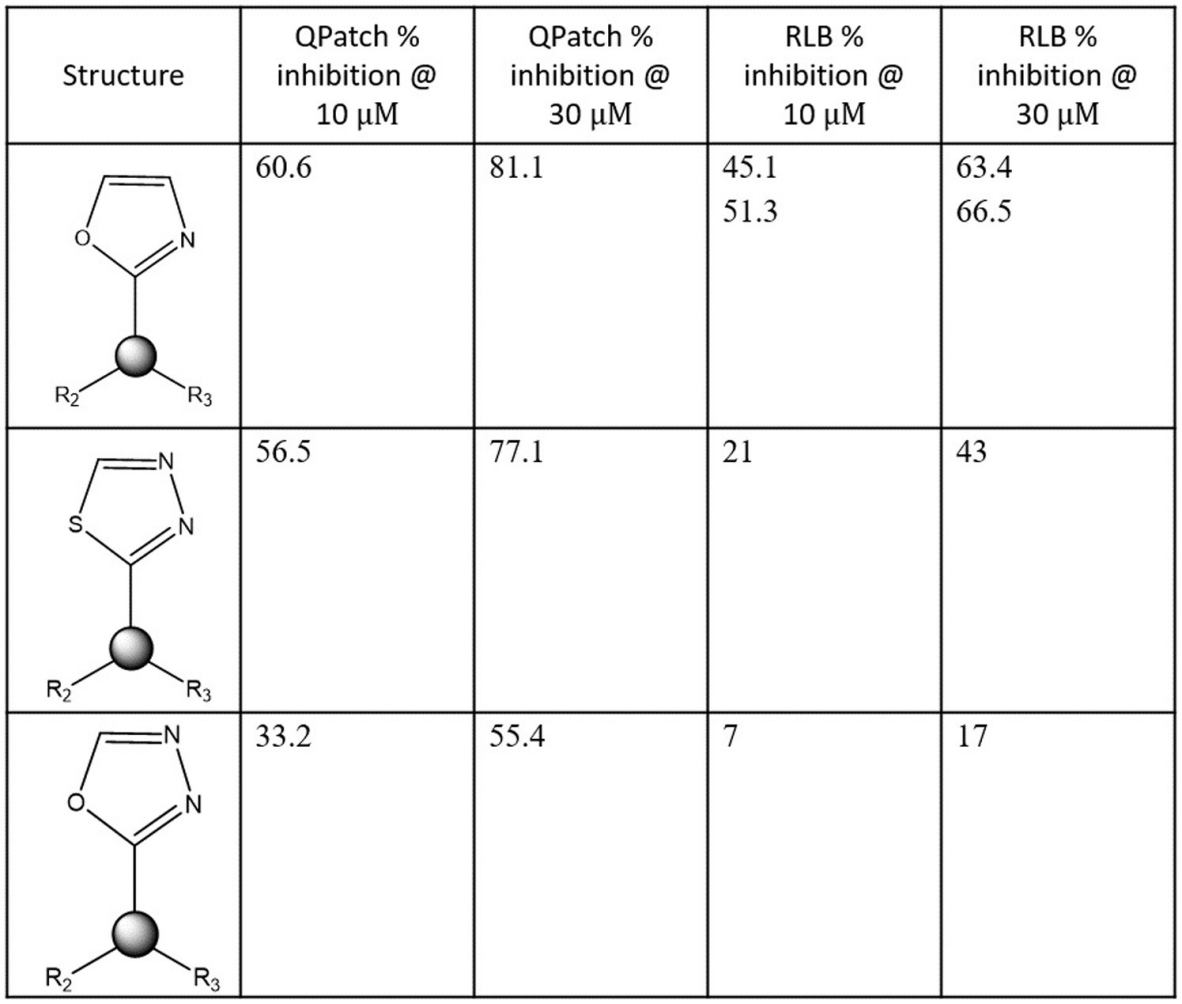
Representative activity cliffs for the proprietary hERG blocker series 2 (see text) comprised of R1 (corresponding to BP) and R2/R3 (corresponding to BC). The data suggests that hERG occupancy is disrupted by the projection of a highly polar group (oxadiazole) versus a less polar group (sulfadiazole or oxapyrrole) into the putative localized region of H-bond depleted solvation at site Y.

We inferred certain structure-kinetics relationships from the structure-activity relationships of two proprietary in-house datasets based on the aforementioned principles. A large activity cliff in dataset 1 is attributable to the increased de-solvation cost of the pyridazyl versus pyridyl moieties predicted to bind in site C (Fig 10) and the 1- versus 2-pyridyl combined with Cl versus F (Fig 11). The structure-property relationships underlying the solvation differences among these groups is unobvious. A large activity cliff in dataset 2 can be attributed to increased de-solvation cost of the oxadiazole versus sulfadiazole and oxapyrrole groups predicted to project into a cluster of H-bond depleted solvation in site Y (Fig 12), which putatively manifests as slowed k_on_ due to the loss of H-bonds of polar group solvation transferred to bulk solvent. We note that key local solvation effects may be masked in global changes in scalar logP and solubility. Non-trappable blocker-induced expulsion of this solvation is expected to slow k_off_, minimally to the rate of channel closing (below which channel closing becomes rate-determining). k_off_ slowing in the case of shorter BP groups falling short of the Y site depends on the expulsion of H-bond depleted solvation from the C region (equating to an enthalpic re-solvation loss during dissociation, together with an entropic de-solvation gain during association).

### Proposed blocker structure-trappability relationships

The concept of blocker trappability was proposed by Starmer et al. [40] and Stork et al. [41] (referred to by Armstrong et al. [42] and Mitcheson et al. [43] as the “foot-in-the-door” model). Non-trappable and trappable blockers are differentiable in patch clamp experiments on the basis of channel gating frequency-dependent versus independent inhibition, respectively. Higher frequency stimulation results in proportionately greater fractional open channel time, and therefore, increased binding site accessibility to non-trappable blockers that build and decay during each gating cycle (noting that trappable blockers accumulate to their steady-state occupancy regardless of the open channel time, merely accumulating faster with increasing open time). Windisch et al. studied structure-trappability relationships for a series of analogs around the known trappable blocker, propafenone [29]. We set about to explain this relationship by overlaying and comparing the predicted conformational properties of these analogs vis-à-vis our proposed canonical drain-plug model (see Materials and methods for a description of our conformational analysis approach).). We make the following assumptions:

1. Trappable and non-trappable blockers occupy a common region of P in the open state of the channel (referenced to the parent propafenone moiety of the series).
2. The shape and volume of P differs between the open and closed channel states, which is necessarily localized to the region occupied by the terminal para moiety of the piperidine/piperazine ring of the substituted propafenone analogs (denoted as PT).
3. PT is partially constricted in the closed state of the channel. Trappable blockers are sterically compatible with this putative constriction zone, whereas non-trappables are not (resulting in expulsion of the latter during channel closing).

**Fig 13:**
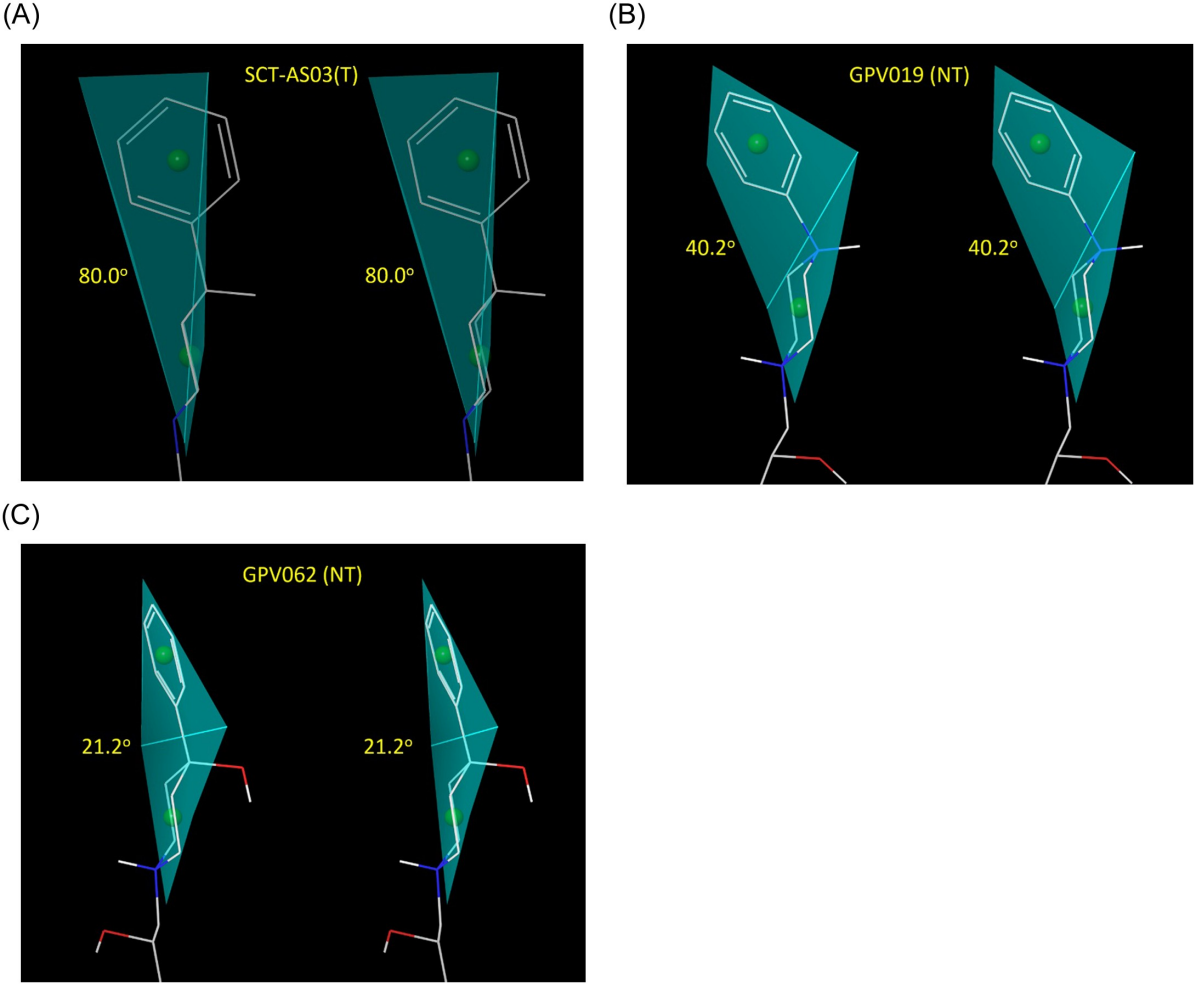
Unambiguous conformational differences are predicted between experimentally identified Trappable (T) and Non-Trappable (NT) propafenone analogs, exemplified by three representative compounds. We hypothesize that in all cases, registration exists between the phenyl group and the putative constriction zone in the closed channel, and furthermore that specific blocker conformational properties are required in this region. (A) The phenyl group of the trappable analog, SCT-AS03, exists in an approximately orthogonal conformation relative to the attached piperidine ring (~80°), resulting in a quasi-linear longitudinal axis in this region that putatively coincides with the pore axis in the bound state. (B) The phenyl and piperazine groups of the non-trappable analog GPV019 are relatively coplanar, and tilted off-axis by ~40° (putatively projecting away from the pore axis in the bound state). (C) A similar coplanar relationship exists between phenyl and hydroxy-substituted piperidine in the non-trappable analog GPV062, which is likewise tilted off-axis by ~20°.

**Fig 14:**
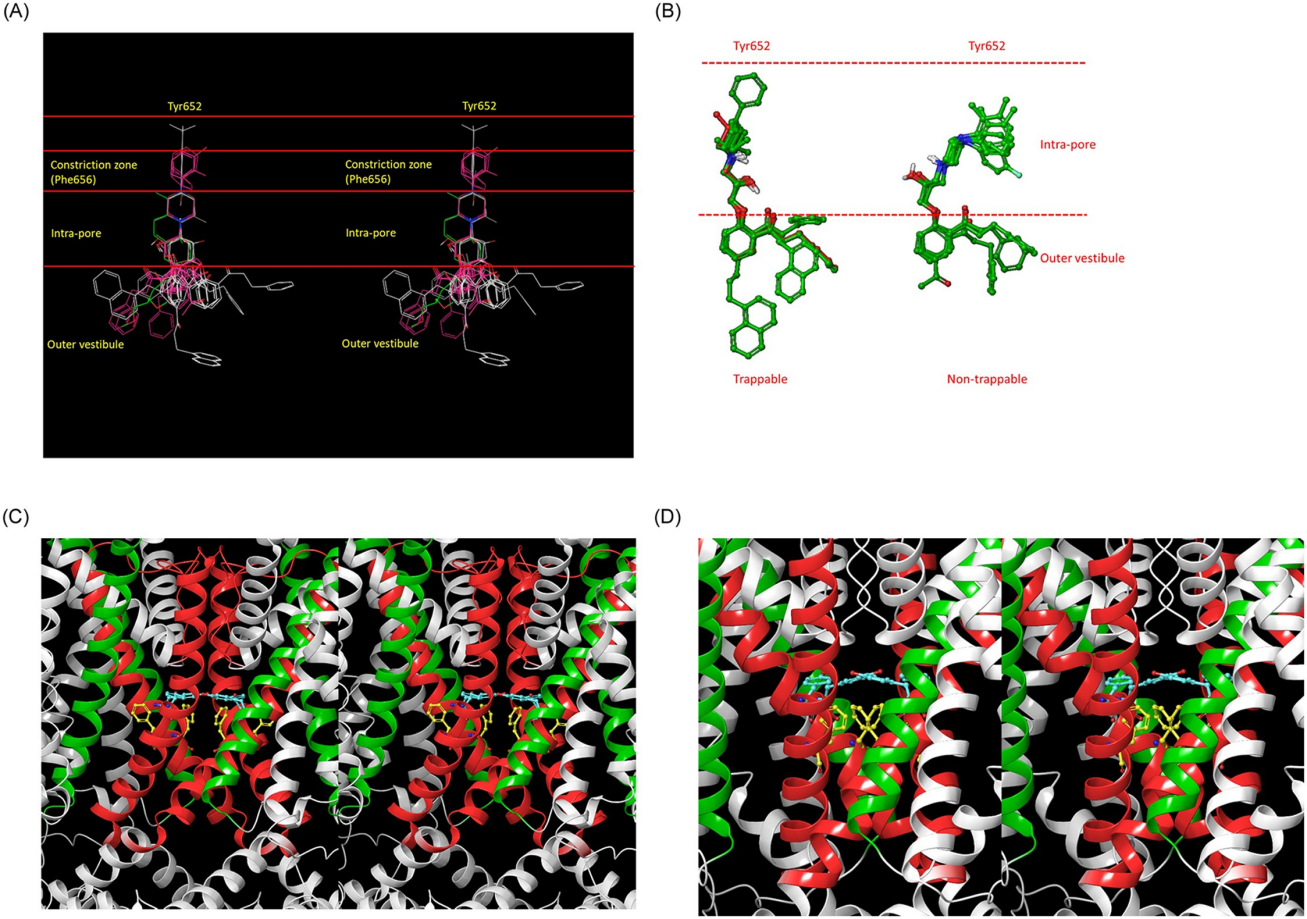
Our blocker overlay model (Fig 3) mapped to the various zones of the channel, including the putative trappability-determining constriction zone that forms during channel closing, from which non-trappable blocker moieties are sterically excluded. (A) Stereo view of the blocker overlay. All of the trappable propafenone analogs either terminate prior to this zone, or project a planar group into it (magenta) that coincides with the pore axis. (B) Zoomed-in view of the putative blocker constriction zone, demonstrating the non-planar conformations of non-trappable blockers in this region. (C) The open constriction zone, comprised of Phe656 (yellow side chains) in hERG, with Tyr652 shown for reference (cyan side chains). (D) The closed constriction zone, comprised of Phe468 (yellow side chains), with Tyr464 shown for reference in EAG1 (cyan side chains).

It then follows that:

1. Steric compatibility between PT and trappable blockers depends on:
  - Occupation by a planar moiety (e.g. phenyl group).
  - Alignment of the planar moiety with the pore axis, as determined by the geometric relationship between the planar moiety and the moiety to which it is bonded (e.g. the aryl-piperidine/piperazine substructure of SCT-AS03, GPV574, GPV031, GPV019, GPV062, and GPV576 shown in Fig 2).
2. Trappability of SCT-AS03 is defined unambiguously by an approximately orthogonal relationship between the planes of the piperidyl and phenyl rings, together with an approximately linear long axis of the phenylpiperidyl substructure (Fig 13A) (noting that the slight rotation of the phenyl ring observed in the Figure is likely due to inaccuracy of the calculated minimum energy conformation). Non-trappability of GPV019, GPV062, and other analogs is defined by an approximately parallel relationship between the same two rings, together with kinking of the long axis of the substructure (Fig 13B and 13C, respectively). The relative positioning of the p-phenyl moiety among the analogs of our overlay is shown in Fig 14A and 14B.

We hypothesize that the putative constriction zone is comprised of Phe656 side chains that rearrange from peripheral positions observed in the cryo-EM structure to more central positions during channel closing. This hypothesis is supported by the observed position of Phe468 in the cryo-EM structure of EAG1 (corresponding to Phe656 in hERG) (Fig 14C and 14D).

In summary, blocker binding is driven largely by:

1. Concurrent steric shape complementarity to the C (L- or Y-shaped blocker moieties), P (quasi-linear blocker moieties), and Y regions of the channel.
2. Principally, blocker de-solvation costs during translocation from the outer vestibule into the C, P, and Y (optionally) regions of the channel.
3. Principally, channel re-solvation costs at the Y and C sites incurred during blocker dissociation from P.
4. Electrostatic interactions with basic blockers that are capable of projecting their charged group along the longitudinal axis of P (Figs 6 and 7, right).

Trappable blockers either terminate within P prior to the putative constriction zone (denoted as PT) formed by Phe652 side chains, or project a planar group into PT along the pore axis. Trappable blockers accumulate occupancy over multiple channel gating cycles (i.e. heartbeats), peaking at the intra-cellular free C_max_, whereas non-trappable blocker occupancy builds and decays within the ventricular hERG population during each cycle [21]. The maximum dynamic occupancy (corresponding to k_off_/k_on_) across all cycles within a dosing period peaks at the free intra-cellular C_max_ for both trappable and non-trappable blockers. Trappable blocker occupancy is agnostic to channel gating frequency (i.e. on-rate merely governs the rate of buildup to the maximum occupancy) whereas non-trappable blocker occupancy depends on k_on_ and k_off_ relative to the channel opening and closing rates, respectively (which has important implications for *in vitro* hERG assays).

### Possible deficiencies of the *status quo* hERG safety assessment protocol

The Redfern SI (eq 1) was derived from a comparison of measured hERG IC_50_, therapeutic C_max_, and maximum observed C_max_ for 100 marketed drugs classified according to TdP propensity: withdrawn drugs versus multiple reported cases, versus isolated cases, versus no reported cases, versus anti-arrhythmic hERG blocking drugs. We have identified the following potential deficiencies in the derivation and application of this widely used metric:

#### Deficiency 1

Substitution of eq 1 into the Hill equation yields a maximum safe hERG blocker occupancy (*γ*_1_) = 3% [21] (by all accounts, a highly stringent safety margin in the absence of underlying cardiovascular disease):

$$\gamma_{1}=\frac{{\left[blocker\right]}_{free(cell)}}{{\left[blocker\right]}_{free(cell)}+K_{d}}=\frac{{\left[blocker\right]}_{free(cell)}}{{\left[blocker\right]}_{free(cell)}+30\cdot {\left[blocker\right]}_{free(cell)}}=\frac{{\left[blocker\right]}_{free(cell)}}{31\cdot {\left[blocker\right]}_{free(cell)}}≅3\%$$ (2)

**Fig 15:**
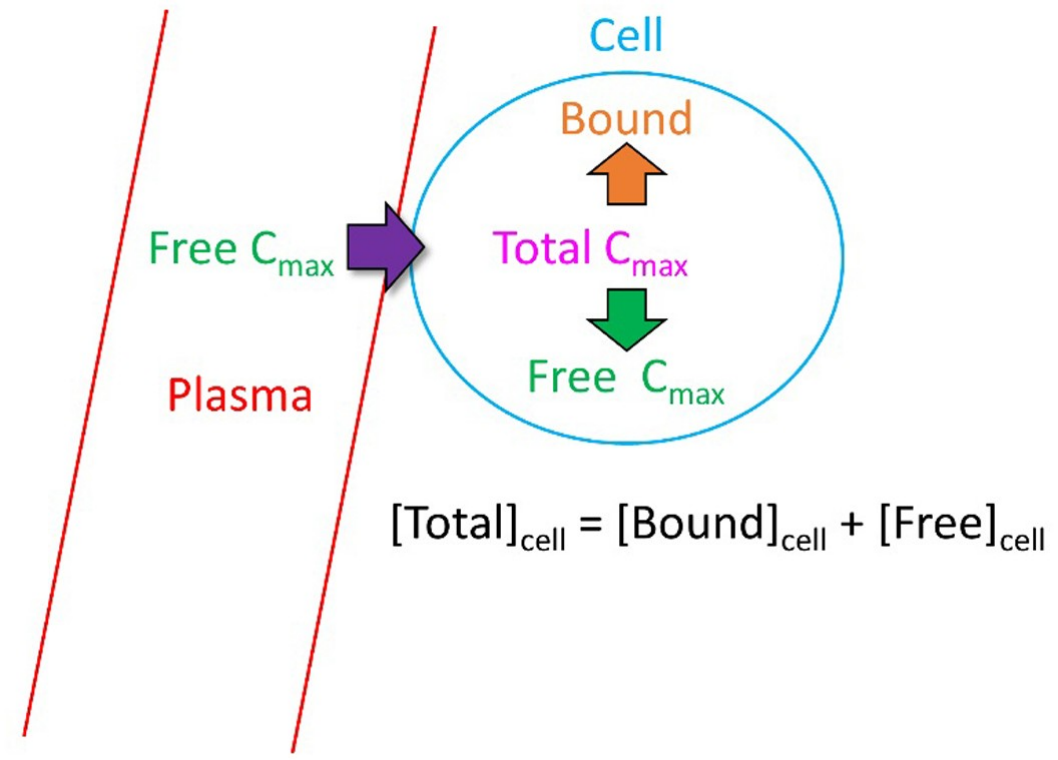
TFPC_max_ equates to TTIC_max_, such that the Hill equation, which is based on free concentration, cannot be solved (see text).

However, it is apparent that the TFPC_max_ equates to the therapeutic total intra-cellular C_max_ (hereinafter referred to as TTIC_max_) (Fig 15). Substitution of [blocker]_free(cell)_ by [blocker]_total(cell)_−[blocker]_bound(cell)_ into eq 3 results in an under-determined equation in the absence of [blocker]_bound(cell)_ information:

$$\gamma_{2}=\frac{{\left[blocker\right]}_{total(cell)}-\;{\left[blocker\right]}_{bound(cell)}}{{\left[blocker\right]}_{total(cell)}-\;{\left[blocker\right]}_{bound(cell)}+K_{d}}$$ (3)

However, the ratio of *γ*_2_ to *γ*_1_ is necessarily less than 1 because [blocker]_total(cell)_ in eq 3 is always greater than [blocker]_free(cell)_:

$$\frac{\gamma_{2}}{\gamma_{1}}=\frac{31\cdot {\left[blocker\right]}_{free(cell)}}{{\left[blocker\right]}_{free(cell)}+30\cdot {\left[blocker\right]}_{total(cell)}}<1$$ (4)

For example, at a [blocker]_total(cell)_ = 5 [blocker]_free(cell)_:

$$\frac{\gamma_{2}}{\gamma_{1}}=\frac{31\cdot {\left[blocker\right]}_{free(cell)}}{151\cdot {\left[blocker\right]}_{free(cell)}}≅0.2$$ (5)

Viewed in this light, the Redfern SI equates to approximately zero safe hERG occupancy at the TFPC_max_ (= TTIC_max_), which serves to buffer exposure and occupancy escalation between the TFPC_max_ and arrhythmic FPC_max_ stemming from DDI, overdose, and other factors. A wide safety margin of this nature is arguably justified given the life and death implications of drug-induced TdP (exacerbated in the presence of pre-existing cardiac impairment), although possibly tempered by questionable assumptions in the Redfern SI derivation.

#### Deficiency 2

**Fig 16:**
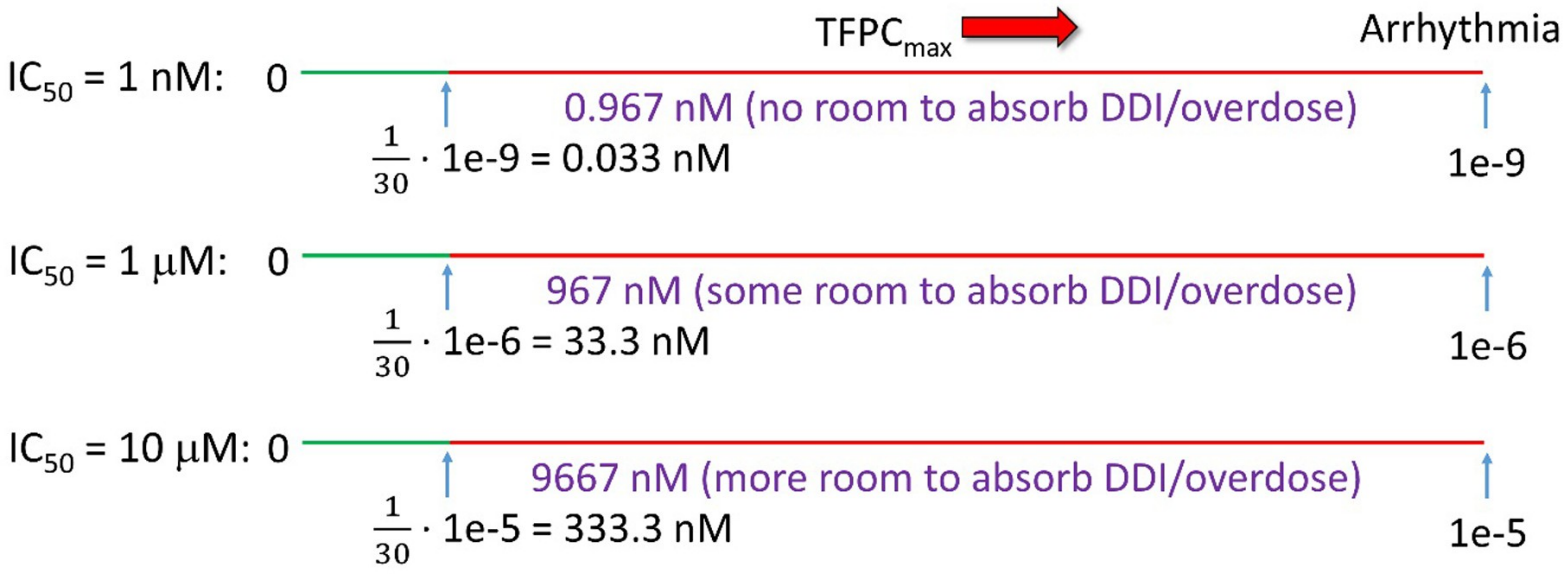
The Redfern et al. derivation is tacitly based on the assumption that the hERG safety margin consists of the exposure range between the TFPC_max_ and the maximum FPC_max_ due to DDI, overdose, or other factors, equating to TFPC_max_ ≤ $\frac{1}{30}$ hERG IC_50_. For the present purpose, we assume that arrhythmia (the primary origin of organ-level TdP) occurs at ~50% block of the open hERG population in otherwise normal M cells (corresponding to the apparent IC_50_), and possibly much lower in the diseased heart, translating to an absolute Δ(exposure)-based Redfern SI = hERG IC_50_ –hERG IC_50_/30.

Both the TFIC_max_ (which correlates with the TFPC_max_) and hERG safety margin necessarily translate to a maximum FIC_max_ (at both therapeutic and escalated exposures due to DDI or overdose) far below our predicted arrhythmic FIC_max_ (AFIC_max_) in the undiseased heart. The hERG IC_50_/30 criterion derived by Redfern et al. [44] is implicitly weighted toward the most potent TdP-inducing drugs in the dataset, which correctly accounts for the narrow exposure window between the maximum observed FIC_max_ and AFIC_max_. However, this relationship breaks down with increasing hERG IC_50_ because the absolute exposure window (i.e. Δ(exposure) = arrhythmic FIC_max_−hERG IC_50_/30) matters, rather than the ratio. In our previous work, we used the O’Hara-Rudy model to predict the cellular arrhythmic threshold of hERG inhibition relative to the open and total hERG populations in otherwise normal midmyocytes (M cells), which varies with cell type (M cells being the most sensitive by far) and blocker trappability [21]. Here, we assume a more conservative estimate of ~50% blockade of the open/conducting hERG population (translating to ~50% reduction of I_Kr_ at a free intra-cellular exposure ≈ hERG IC_50_), noting that we are currently re-exploring hERG blocker dose-response behavior (results to be reported elsewhere). The Redfern SI can thus be roughly expressed as: Δ(exposure) = hERG IC_50_ –hERG IC_50_/30, which is exemplified in Fig 16 for hERG IC_50_ = 1 nM, 1 μM, and 10 μM. It is apparent from these examples that the upper safe TFPC_max_ in otherwise normal M cells is greatly underestimated for μM potency blockers by the ratio-based one-size-fits-all Redfern SI, compared with absolute Δ(exposure).

#### Deficiency 3

Blocker occupancy (proportional to percent inhibition) is channel state- and time-dependent, and therefore out of the scope of eq 3:

$$\gamma (t)=k_{on}\cdot {\left[blocker\right]}_{free}(t)\cdot (P_{o}+P_{i})\cdot (1-\gamma (t))-k_{off}\cdot {\left[blocker\right]}_{free}(t)\cdot (P_{o}+P_{i})\cdot \gamma (t)-j\cdot (\beta \cdot P_{o}+\mu \cdot P_{i})\cdot \gamma (t)$$ (6)

where [*blocker*]_*free*_(t) is the time-dependent free intra-cellular blocker concentration, *P*_*o*_ *and P*_*i*_ are the open and inactive state probabilities, *β* is the channel opening rate constant, and *μ* is the channel inactivation rate constant [21]. Non-trappable blocker occupancy at a given [*blocker*]_*free*_ is overestimated by eq 3 when k_on_ is less than the channel opening rate, and [*blocker*]_*free*_ is below the saturating level. Trappable blocker occupancy is described by eq 3 when k_off_ ≥ k_off_ floor (which cannot be ascertained from IC_50_ measurements alone). The *in vivo*-relevant percent inhibition for both trappable and non-trappable blockers is ideally measured via manual patch clamp experiments performed at the physiological gating frequency. Given the fast rate of channel opening (the peak amplitude is reached in a few ms), the k_on_ requirement for high percent inhibition among non-trappable blockers is necessarily fast [21]. Zu et al. measured the rate constants for several hERG blockers (including some listed in Fig 1) in static channels via a radio-ligand displacement approach [45]. Using cisapride (a known non-trappable blocker) as a benchmark, we hypothesize that all of the measured rate constants in the authors’ dataset are up to 1,000-fold slower relative to physiological conditions (noting that k_on_ and k_off_ were deconvoluted by the authors from the measured k_obs_ = k_on_ [blocker]_free_ + k_off_ and the IC_50_).

#### Deficiency 4

**Fig 17:**
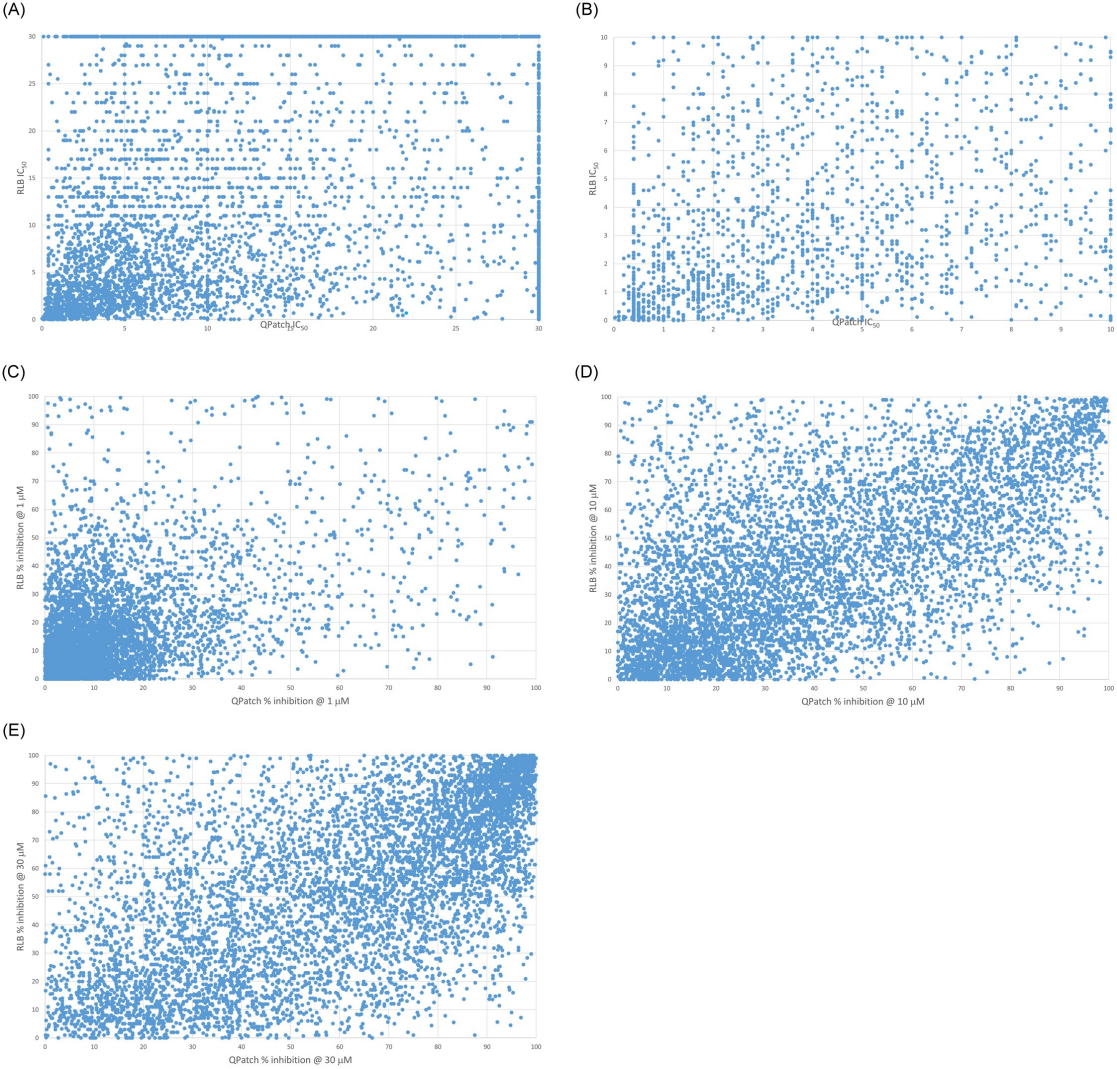
Plots of measured high throughput QPatch versus radio-ligand binding (RLB) assays for 7,231 internal proprietary compounds. (A) IC_50_. (B) IC_50_ zoomed to the 0 to 10 μM range. (C) Percent inhibition @ 1 μM. (D) Percent inhibition @ 10 μM (E) Percent inhibition @ 30 μM. The lack of agreement between the two assays at all percent inhibition levels is apparent. The accuracy/sensitivity of this data (i.e. the ability to resolve true structure-activity relationships) may be insufficient for a number of reasons (including solubility and permeability limitations) to support SAR analysis needed for guiding hERG mitigation, as well as for constructing QSAR and machine learning models.

*Status quo* hERG mitigation and preclinical safety assessment is typically guided by high throughput *in vitro* IC_50_ data and animal PK data. hERG mitigation depends on guidance from accurate data capable of unambiguously resolving the true SAR [46], which is difficult to measure via high throughput *in vitro* experimental methods (Fig 17) (noting the often flat hERG free energy landscape across primary target potency-sparing chemical series). Mitigation is aimed minimally at satisfaction of the Redfern SI, and maximally at total abrogation of hERG activity (i.e. IC_50_ > the detectability limit) among clinical drug candidates. However, total hERG mitigation, combined with optimal primary target potency and other requirements, is rarely achieved in practice, relegating high uncertainty to both terms of the Redfern IC_50_/exposure ratio. Uncertainty on top of uncertainty is especially problematic for safety assessment of clinical candidates in advance of *in vivo* cardiotoxicity evaluation.

### Proposed clinically-relevant hERG safety assessment and mitigation approaches

**Fig 18:**
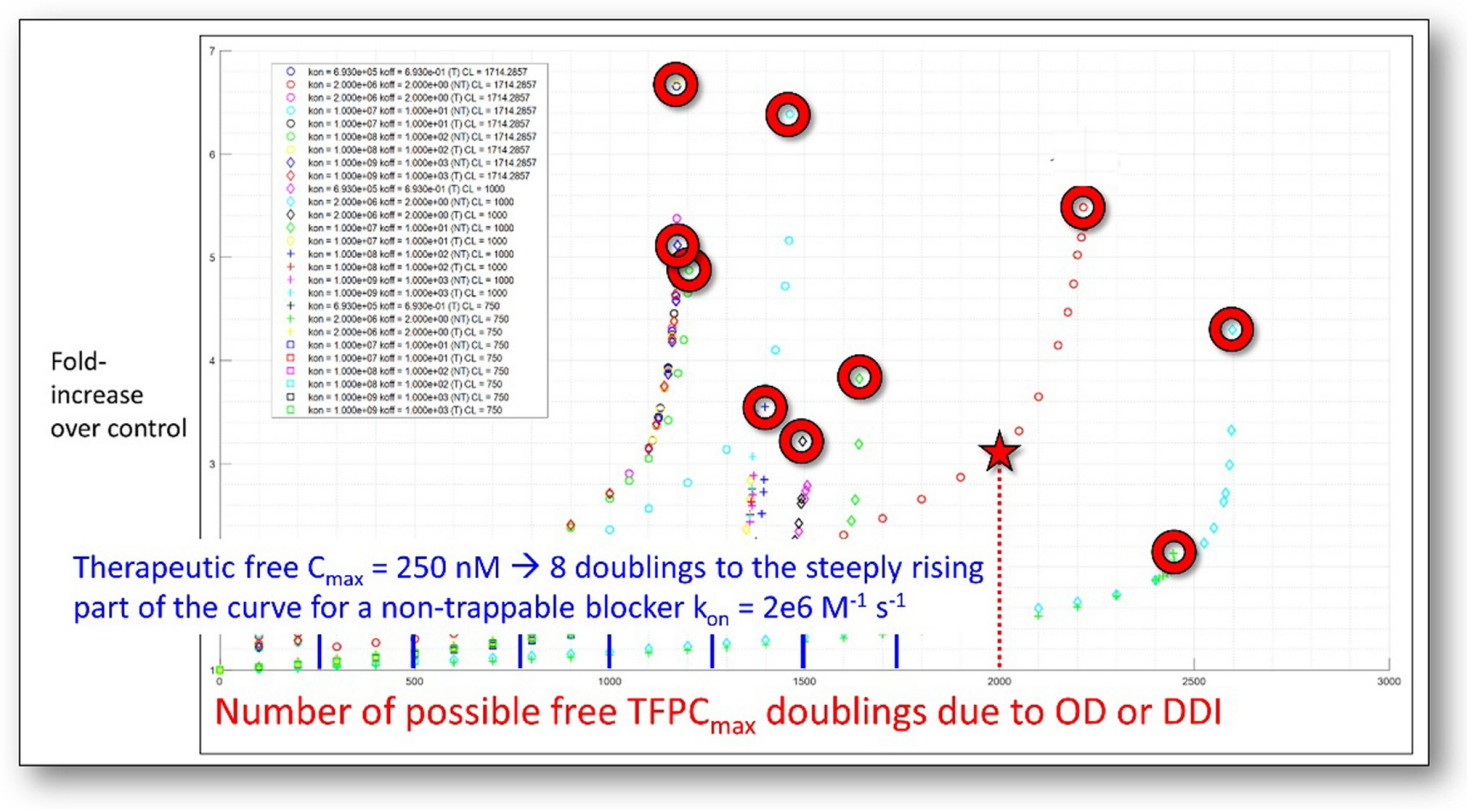
Hook-shaped dose-response curves for trappable and non-trappable blockers simulated using our modified version of the O’Hara-Rudy model of the undiseased human heart as a function of k_on_, k_off_, and hERG IC_50_. The hollow circles denote the “pre-arrhythmic” tipping-point exposure at the precipice of the arrhythmic response. The hERG safety margin should reside somewhere on the quasi-linear region of the corresponding curve (far from the tipping point) within a certain number of exposure doublings between the TFPC_max_ and tipping point, commensurate with the benefit/risk of the disease indication (zero hERG blockade at the TFPC_max_ is of course ideal). The safety margin decreases with increasing percent inhibition at the TFPC_max_, but potentially more slowly than suggested by the Redfern SI.

As addressed above, pro-arrhythmic risk at exposures ≥ the TFPC_max_ in humans is putatively overestimated by the *in vitro* IC_50_-based Redfern SI in patients with uncompromised cardiac function (a key assumption in our simulations). Uncertainty in the hERG safety of late stage lead compounds exhibiting residual activity is often further complicated by the lack of confirmed quantitative hERG data (which may extend to clinical candidates in some cases). Instead, we propose the following guidelines:

1. Minimizing TFPC_max_ via kinetically tuned drug-target binding, defined as the optimization of k_on_ to the rate of binding site buildup (explained in detail in [21] and [22]).
2. Testing for blocker trappability based on the structural criteria outlined above (Figs 13 and 14), together with frequency-independent percent inhibition, using an appropriate patch clamp protocol [41].
3. Wherever possible, performing trappable → non-trappable chemical transformations based on the structural guidance outlined above, (noting that non-trappable blocker occupancy does not accumulate over time).
4. Basing SAR and safety assessment on percent inhibition, rather than IC_50_ data (noting that error may be amplified in best-fit dose-response curves, that percent inhibition is a more direct measure of blocker-hERG occupancy at concentrations of interest; and prediction of fractional hERG occupancy under *in vivo* conditions from IC_50_ depends on knowledge of TFIC_max_, rather than TFPC_max_). Sufficient accuracy is needed to confirm critical SAR and inform safety assessment. Data accuracy can be gauged via the following criteria:
  - Similarity in observed trends between orthogonal assays (e.g. patch clamp and radio-ligand binding).
  - Convergence of replicate runs in each assay.
  - Self-consistency of hERG SAR (the existence of identifiable hERG structure-activity drivers across a given chemical series).
5. Increasing the de-solvation cost (reflected in the polarity [38,39]) of the proposed BC, BP, and BY features in order to slow k_on_ (especially BY, when present), while maintaining on-target activity, etc.
6. Attenuating the pKa of basic group(s) in BP (when unavoidably present) in order to slow k_on_ and minimize undesirable collateral lysosomal trapping and membrane partitioning effects manifesting as high volume of distribution (V_ss_).
7. Maximally decreasing percent hERG inhibition at the projected TFPC_max_ (corresponding to the TTIC_max_ adjusted for lysosomal/membrane/off-target binding).
8. Assessing pro-arrhythmicity based on Δ(exposure) between the projected TFPC_max_ and hERG IC_50_ (noting that *in vitro* potency may be overestimated for non-trappable blockers). Our simulated general dose-response relationship for trappable and non-trappable blockers as a function of k_on_ and k_off_ (for non-trappables) and IC_50_ (for trappables) is shown in Fig 18 (simulated using the O’Hara-Rudy model of the undiseased human heart [21,47]).
9. Attenuating blockade of other cardiac channels (when present), noting that hERG blockade in the absence of multiple ion channel effects (MICE) is pro-arrhythmic, as is blockade of other single cardiac ion channels. The emphasis by other workers on MICE [15] may reflect the poor translatability of *in vitro* hERG IC_50_ data to humans, although the normal inward-outward ion current balance is certainly disrupted by MICE (except in the case of verapamil, in which blockade of inward I_Ca_ is believed to offset blockade of outward I_Kr_).

### Chloroquine/hydroxychloroquine case study

**Fig 19:**
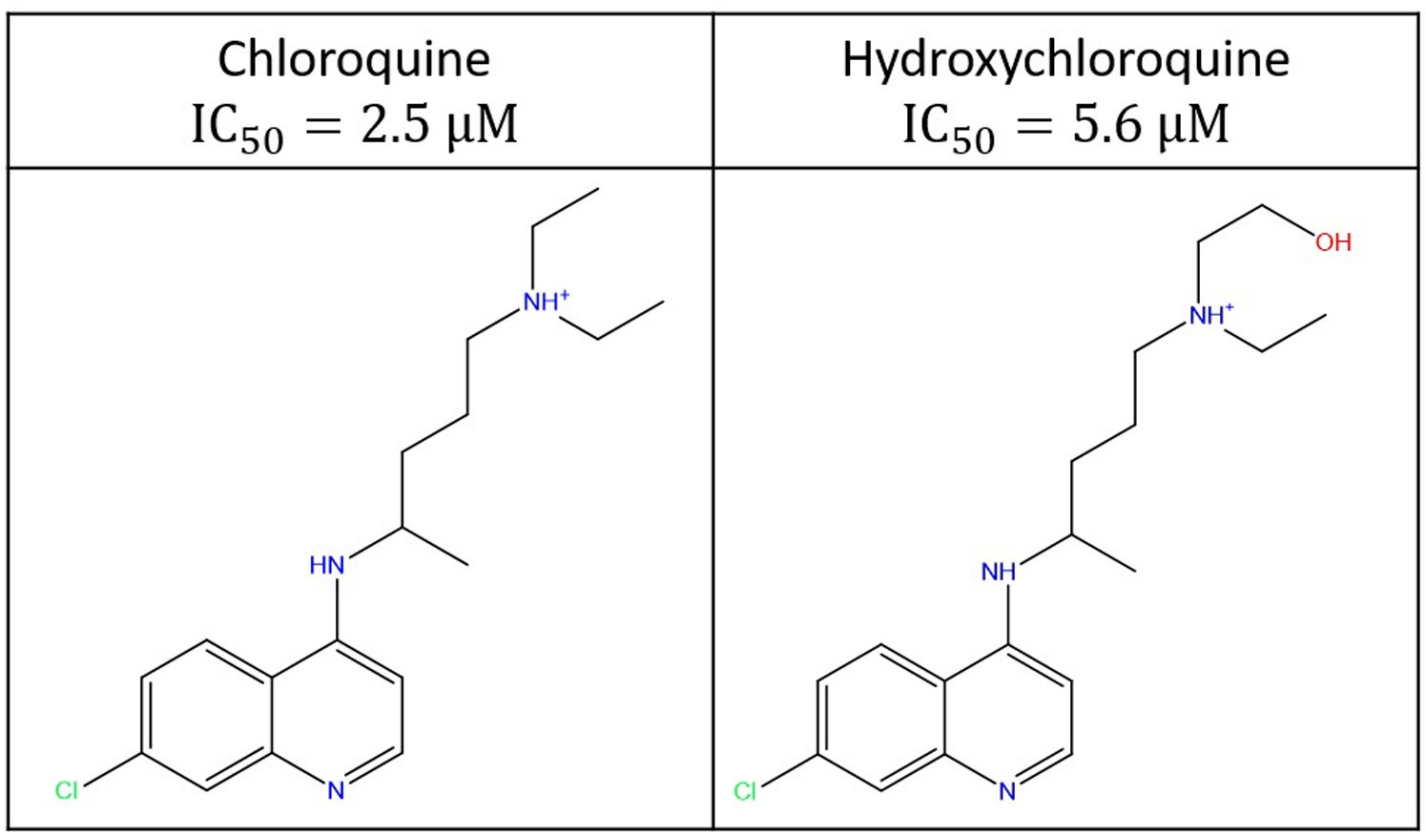
Candidate COVID-19 therapies known to block hERG [54,55], noting that the reported percent hERG inhibition (35%) of hydroxychloroquine (HCQ) at 3 μM was used to estimate the IC_50_ via the Hill equation.

Chloroquine and its hydroxy derivative are currently of interest as potential SARS-CoV-2 anti-viral therapies [48] (Fig 19). The anti-viral efficacy of these drugs is unconfirmed (and is under considerable scrutiny), whereas both drugs are known to exhibit pro-arrhythmic and arrhythmic levels of hERG blockade in the clinical setting (based on data from the Federal Adverse Event Reporting System summarized in [49], a recent COVID-19 patient cohort [50], and reports of QT prolongation in COVID-19 patients [51,52]). Furthermore, hERG is likely blocked additionally by the principal metabolites of HCQ, which consist of desethylchloroquine, desethylhydroxychloroquine, and bisdesethylchloroquine [53].

**Fig 20:**
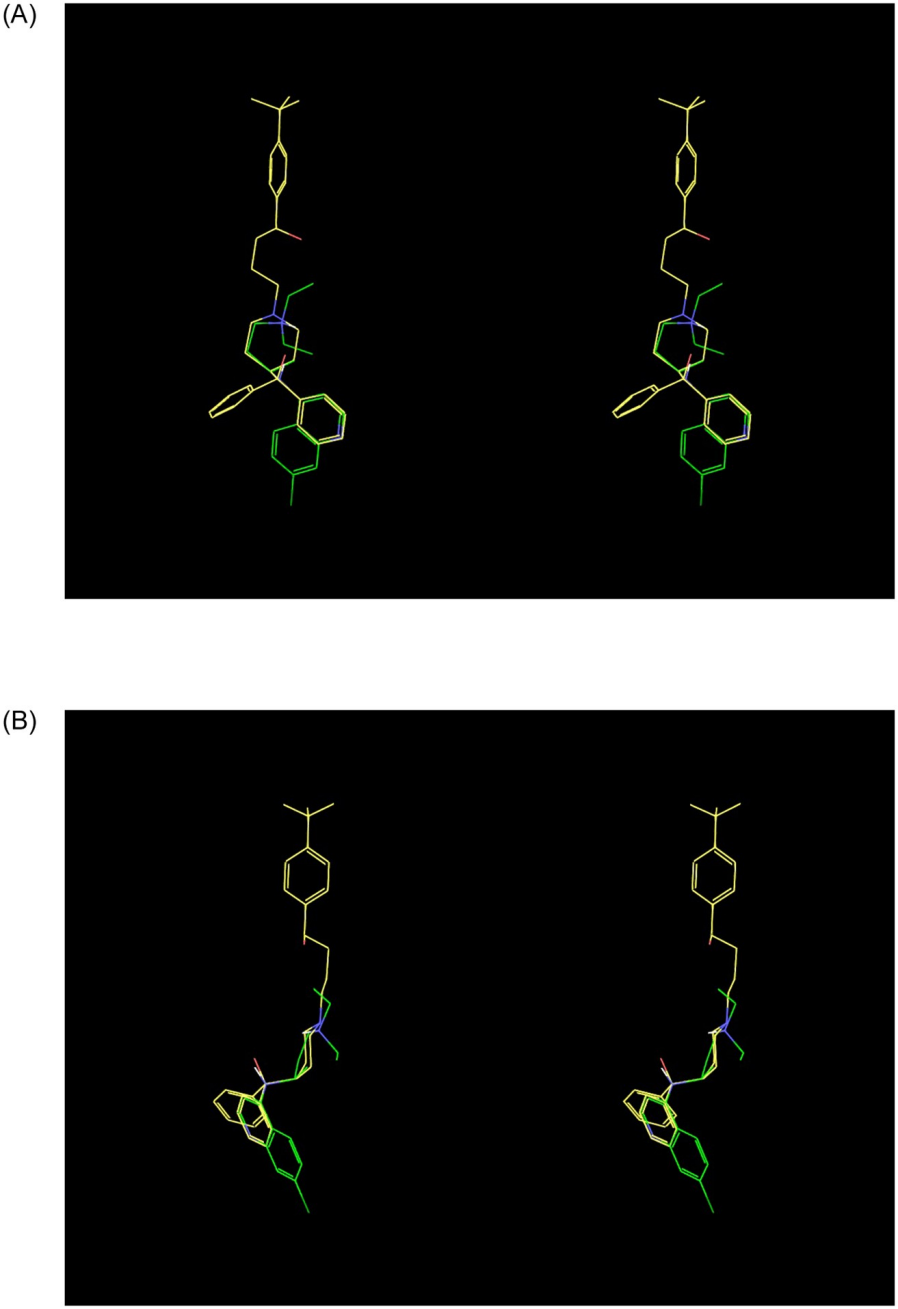
(A) Front view of chloroquine (green carbons) overlaid on our hERG blocker model (terfenadine shown for reference (yellow carbons)). (B) Side view of the overlay shown in A.

The reported hERG IC_50_s for chloroquine and HCQ are 2.5 μM [54] and ~5.6 μM (the latter of which we estimated from the Hill equation based on 35% inhibition at 3.0 μM reported in [55]). Both drugs fit unambiguously to our hERG blocker overlay model (shown for chloroquine in Fig 20), with BP terminating well below the putative constriction zone in the closed channel state in the absence of a BY group (consistent with trappability). We note that the putatively higher IC_50_ of HCQ is consistent with the expected greater incremental de-solvation cost of the hydroxyl group, which is predicted by our model to reside on BP. Neglecting the additive effects of azithromycin or other drug combinations, the maximum safe TFPC_max_ for chloroquine and HCQ under the Redfern SI equates to 83 and 187 nM (i.e. 2.5E-6/30 and 5.6E-6/30 μM), respectively. The mean total plasma concentration of orally administered HCQ in one COVID-19 study is 0.46 μg/ml [48], equating to ~1.4 μM (compared with ~1.2 μM in malaria patients [56]), which is not necessarily representative of all patients. Confirmation of the reported potency and plasma PK of HCQ and its metabolites at therapeutic COVID-19 dosing levels is essential for accurate hERG safety assessment. The high V_ss_ of HCQ and its metabolites [56] results in extremely slow renal excretion (mean terminal t_1/2_ of 40–50 days), raising the possibility of multi-dose accumulation. The estimated TFPC_max_ of HCQ adjusted for an ~50% plasma protein bound fraction [53] is ~700 nM (i.e. 0.5 1.4E-6 μM), or ~3.7-fold above the Redfern SI (i.e. 700/187). The extent to which the TI may be underestimated by the Redfern criterion depends on the true FIC_max_ in humans (noting that the fractional hERG occupancy of trappable blockers accumulates to FIC_max_/(FIC_max_ + hERG IC_50_). Chloroquine and HCQ lack definitive preclinical hERG safety margins vis-à-vis the Redfern criterion, which is exacerbated by putative hERG trappability and low plasma protein binding (possibly traded against high lysosomal uptake [57]). HCQ has nevertheless realized many prescription-years as an anti-malarial [58] and auto-immune therapy, the safety profile of which is well-understood for those specific indications. All safety indices are context dependent, and can be exceeded (and the TI lost) in cases of significant exposure escalation above the established therapeutic level due to additive multi-drug effects, and/or the impact of underlying disease on drug clearance or the safety threshold. LQT monitoring and exposure control are essential for off-label HCQ administration, given:

1. That arrhythmia can result from even transient excursions in exposure ≥ the arrhythmic threshold (analogous to a “spark”). The high potential for cardiovascular impairment in COVID-19 patients may result in a downward shift of our predicted arrhythmic hERG occupancy level (which was predicted from simulations of the undiseased human ventricular cardiomyocyte).
2. Co-administration with other pro-arrhythmic drugs, including azithromycin (hERG IC_50_ = 219 μM [59]), may lower the safe exposure level of HCQ (noting that reported cases of azithromycin-induced arrhythmia [49] have been attributed to intra-cellular Na^+^ loading [59]).
3. That HCQ metabolites likewise block hERG and exhibit long t_1/2_.
4. The high potential for impaired clearance due to renal compromise in COVID-19 patients.
5. The potential for DDIs in COVID-19 patients undergoing multi-drug therapies.
6. That intravenous HCQ administration results in up to 19-fold higher blood levels (2,436 ng/ml [56], translating to ~13 μM) compared with oral administration. We note that this level is more than 2-fold above our estimated hERG IC_50_, equating to ~67% inhibition (well above our predicted arrhythmic level).

## Discussion

We used non-equilibrium structure-free energy (Biodynamics) principles, combined with a three-dimensional ligand-based alignment of a set of trappable and non-trappable hERG blockers and published cryo-EM structures of hERG and Na_v_1.4 [30,31], to understand the non-equilibrium structure-free energy relationships governing blocker-hERG binding. Specifically, we propose that mutual blocker-hERG de-solvation and re-solvation costs that respectively govern k_on_ and k_off_ are localized to a set of blocker-specific docking interfaces (denoted as C-BC, P-BP, and Y-BY) in the C-linker and pore cavities. This hypothesis is consistent with our previous ligand-based analysis of hERG blocker chemical space (based on the Redfern dataset [44] and internal patch clamp and radio-ligand binding data), including neutral bisaryl, basic bisaryl, and alkylamine-containing scaffolds [21].

hERG safety assessment and mitigation are necessarily weighted toward the prevention of false negatives over false positives (erring on the side of caution), which is entirely justified given the acute, life-threatening implications of TdP, combined with:

1. Uncertainty in the true cause-effect relationship between measured hERG inhibition and arrhythmic risk.
2. The chicken-egg nature of hERG assessment/mitigation during the lead optimization stage, stemming from the lack of *in vivo* ECG and PK data needed to establish a human-relevant safety margin. Critical unknown parameters during this stage include:
  - The predicted TFPC_max_ in humans.
  - The predicted TFIC_max_ at the TFPC_max_ in humans, noting that percent hERG inhibition cannot be assessed from IC_50_s in the absence of TFIC_max_ information. Furthermore, TFIC_max_ may be influenced by potentially unquantifiable lysosomal trapping, membrane binding, primary target binding, and off-target binding.
  - Confirmed hERG IC_50_ and percent inhibition data, noting that such data is typically measured using less accurate high throughput techniques (Fig 17), and furthermore, may overestimate the dynamic occupancy of non-trappable blockers.

Simultaneous satisfaction of the highly stringent Redfern criterion (translating approximately to zero tolerated hERG inhibition at the TFPC_max_) and efficacious therapeutic target binding criteria is extremely challenging, time-consuming, and failure prone. The question is whether absolute hERG safety at all possible exposures, and across all indications and patient populations is an acceptable tradeoff against slowed progress and failure among hERG-afflicted R&D programs addressing unmet medical needs (which our findings can only help inform, but not answer).

**Fig 21:**
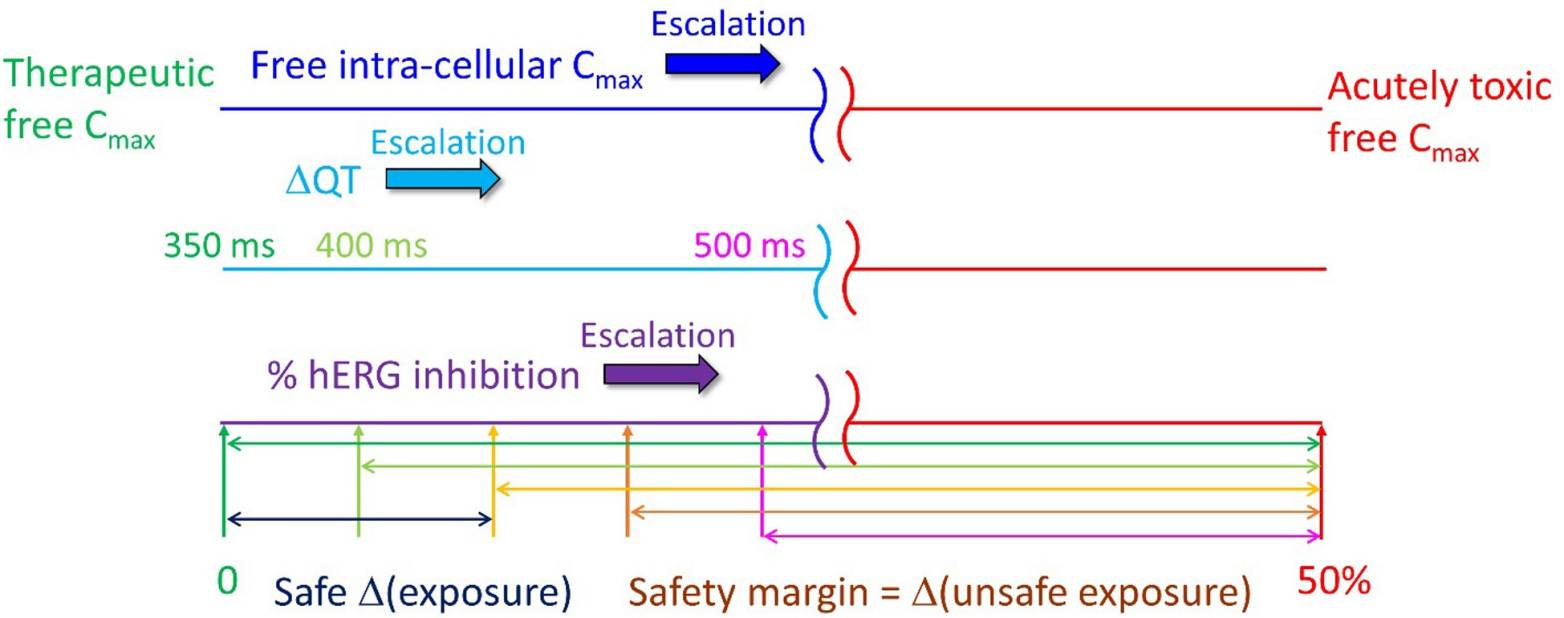
Our proposed safety margin is defined in terms of the fractional hERG occupancy at the therapeutic intra-cellular free C_max_ relative to that at the predicted arrhythmic exposure level in the otherwise normal ventricular cardiomyocyte. This metric differs from the Redfern SI, in which percent hERG inhibition is restricted to effectively zero at the TFPC_max_ (corresponding to the bright green double-headed arrow in the lower part of the figure). The safe Δ(exposure) depends on the benefit/risk of the disease indication, but should always correspond to hERG occupancy far below the arrhythmic level, and ideally zero (assumed here to be ~50% of the open/conducting channel population [21]), together with the QT interval < 500 ms under *in vivo* conditions.

We predicted arrhythmogenic propensity in terms of fractional occupancy of the ventricular hERG channel population in the undiseased human heart at exposures ranging between the TFIC_max_ and maximum FIC_max_ expected from DDIs or overdose (noting that the possible need for dose escalation during clinical trials must be considered in establishing the hERG SI). We define the human-relevant SI in terms of the Δ(exposure) between the true TFIC_max_ (typically > the efficacious free C_max_, allowing for metabolic clearance) and the arrhythmic exposure, which may be significantly less in the presence of cardiac dysfunction. The actual safety margin depends on the maximum Δ(exposure) due to PK variability, DDIs, overdose, or dose escalation (Fig 21). In contrast, the Redfern SI begins with near zero tolerated hERG inhibition at the TFPC_max_, translating to a 100% safety margin relative to the putative arrhythmic blockade level. Furthermore, the Redfern SI appears biased toward potent trappable blockers, which based on our previous analysis [21], demand a greater safety margin than non-trappables.

## Conclusions

In this work, we emphasize the need for human-relevant hERG safety prediction and mitigation criteria during the preclinical stages of drug discovery, accounting for the true relationships between chemical structure and *in vivo*-relevant dynamic hERG occupancy, and between dynamic occupancy and the pro-arrhythmic effects thereof on the otherwise normal human AP in ventricular cardiomyocytes. Both relationships seem to be poorly understood under the conventional wisdom, including reliance on static equilibrium binding metrics and near zero tolerance for blockade at therapeutic exposures. We propose that blocker association is driven by steric shape complementarity, blocker de-solvation cost at key docking interfaces (together with electrostatic attraction in the case of basic compounds), and dissociation is driven largely by mutual blocker and binding site re-solvation costs (noting that the dissociation rate of non-trappable blockers under physiological conditions is necessarily ≥ the rate of channel closing). We further propose a drain-plug-like canonical binding mode, in which blockers straddle the pore and C-linker cavities (analogous to the binding mode of GDN in the cryo-EM structure of Na_v_1.4 [31]), projecting R-groups into non-bulk-like solvation sites in the C-linker (C/BC), and pore (P/BP, Y/BY) regions of the outer vestibule. We attribute trappability to a putative constriction zone that forms during channel closing, with which only planar blocker groups capable of aligning with the pore axis (or the absence of groups at this position) are sterically compatible. Disruption of blocker binding is putatively achievable via trappable → non-trappable chemical transformations, together with incorporation of polar groups at BC, BP, and BY, thereby increasing the blocker de-solvation cost at those sites. We showed that the Redfern SI equates effectively to zero hERG occupancy at the TFPC_max_, and is weighted heavily toward the prevention of false negative blockers from entering clinical trials at the possible expense of false positives. Our approach, guided by accurately measured *in vitro* percent inhibition and human-relevant *in vivo* PK data, may help to minimize trial-and-error mitigation, and lower uncertainty in human-relevant hERG safety predictions.
